# Energetic and genomic potential for hydrogenotrophic, formatotrophic, and acetoclastic methanogenesis in surface-expressed serpentinized fluids of the Samail Ophiolite

**DOI:** 10.3389/fmicb.2024.1523912

**Published:** 2025-01-31

**Authors:** Alta E. G. Howells, Lilja M. Quinn, Miguel G. Silva, Kylie Akiyama, Lucas M. Fifer, Grayson Boyer, Srishti Kashyap, Kirt Robinson, Jared Broddrick, Everett L. Shock, Tori M. Hoehler

**Affiliations:** ^1^NASA Ames Research Center, Moffett Field, CA, United States; ^2^Blue Marble Space Institute of Sciences, San Francisco, CA, United States; ^3^Department of Biology, Washington University, St. Louis, MO, United States; ^4^Department of Aeronautics and Astronautics, Stanford University, Palo Alto, CA, United States; ^5^Department of Bioengineering, University of California, Berkeley, Berkeley, CA, United States; ^6^Department of Earth and Space Sciences, University of Washington, Seattle, WA, United States; ^7^Astrobiology Program, University of Washington, Seattle, WA, United States; ^8^School of Earth and Space Exploration, Arizona State University, Tempe, AZ, United States; ^9^Department of Geological Sciences, University of Colorado, Boulder, CO, United States

**Keywords:** methanogenesis, serpentinization, substrate flux, bioenergetics, genomics

## Abstract

Serpentinization, the reaction of water with ultramafic rock, produces reduced, hyperalkaline, and H_2_-rich fluids that support a variety of hydrogenotrophic microbial metabolisms. Previous work indicates the occurrence of methanogenesis in fluids from the actively serpentinizing Samail Ophiolite in the Sultanate of Oman. While those fluids contain abundant H_2_ to fuel hydrogenotrophic methanogenesis (CO_2_ + 4H_2_ ➔ CH_4_ + 2H_2_O), the concentration of CO_2_ is very low due to the hyperalkalinity (> pH 11) and geochemistry of the fluids. As a result, species such as formate and acetate may be important as alternative methanogenic substrates. In this study we quantified the impact of inorganic carbon, formate and acetate availability for methanogenic metabolisms, across a range of fluid chemistries, in terms of (1) the potential diffusive flux of substrates to the cell, (2) the Affinity (Gibbs energy change) associated with methanogenic metabolism, and (3) the energy “inventory” per kg fluid. In parallel, we assessed the genomic potential for the conduct of those three methanogenic modes across the same set of fluids and consider the results within the quantitative framework of energy availability. We find that formatotrophic methanogenesis affords a higher Affinity (greater energetic yield) than acetoclastic and hydrogenotrophic methanogenesis in pristine serpentinized fluids and, in agreement with previous studies, find genomic evidence for a methanogen of the genus *Methanobacterium* to carry out formatotrophic and hydrogenotrophic methanogenesis, with the possibility of even using bicarbonate as a supply of CO_2_. Acetoclastic methanogenesis is also shown to be energetically favorable in these fluids, and we report the first detection of a potential acetoclastic methanogen of the family *Methanosarcinaceae*, which forms a distinct clade with a genome from the serpentinizing seafloor hydrothermal vent field, Lost City. These results demonstrate the applicability of an energy availability framework for interpreting methanogen ecology in serpentinizing systems.

## 1 Introduction

In the subsurface, where energy from sunlight is not available to life, microorganisms must rely on redox chemical disequilibria as a source of energy. Such disequilibria can result from the reaction of water and rocks. One example is the process of serpentinization, where primary ferrous iron minerals in reduced ultramafic rock from the upper mantle react with water to produce secondary ferric iron minerals and H_2_ (McCollom and Bach, 2009). In addition to being H_2_-rich, fluids generated through serpentinization are reduced and hyperalkaline. H_2_ generated from this process can serve as an electron donor for many microbial metabolisms. When the serpentinized fluid mixes with unreacted fluids rich in electron acceptors, chemical disequilibrium is achieved, and the resulting energy can support life. The energy-rich, habitable zones created in the subsurface by serpentinization are of great interest to astrobiology. Evidence shows that water-rock reactions like serpentinization may occur on the moon of Saturn, Enceladus, and supply H_2_ detected in its plumes (Waite et al., 2017). Water-rock reactions may also be a relevant process for Europa, a moon of Jupiter (Vance et al., 2023). Serpentinizing systems on Earth provide a window into the potential habitability of Enceladus, Europa, and possibly other ocean worlds in our outer solar system. Therefore, of the many subsurface processes that can support life through chemical disequilibrium, we are highly motivated to understand how the subsurface process of serpentinization supports microbial life.

While typically thought of as being associated with the ocean floor along ocean-spreading ridges, serpentinization also occurs where the upper mantle has been uplifted and exposed on the continental crust. One example of continental serpentinization is the Samail Ophiolite in the Sultanate of Oman. Because the Samail ophiolite is located in an arid desert, the fluids receive little influence from surrounding vegetation and the fluid chemistry and associated microbiology are predominantly influenced by water-rock reactions.

The geological and physical processes governing fluid chemistry in Oman are well characterized through geochemical modeling and geochemical and mineralogical characterization (Kelemen et al., 2011; Miller et al., 2016; Canovas et al., 2017; Leong et al., 2021; Nothaft et al., 2021a). In Oman, there are two distinct fluid types. Type II fluids—representative of pristine serpentinized fluids—reacted in the deep subsurface (>500 meters). Type II fluids are highly reduced CaOH_2_ solutions that have high concentrations of H_2_, are hyperalkaline (>pH 11), and have low concentrations of dissolved inorganic carbon (DIC). Type I fluids are partially reacted in the shallow subsurface (<50 meters) and are MgHCO_3_^+^ solutions. Type I fluids are circumneutral (pH 7–8), have a lower concentration of H_2_ than Type II fluids, and are more oxidized due to the influence from the atmosphere (Leong et al., 2021). The two fluid types can mix in the subsurface and at the surface when expressed through fault lines and fissures to create steep geochemical gradients, making the process of serpentinization in Oman an ideal environment to understand the microbiological response to extreme conditions, namely alkaline pH and carbon limitation.

Methanogenesis, particularly hydrogenotrophic methanogenesis (4H_2_ + CO_2_ ➔ CH_4_ + 2H_2_O), has long been considered as a model for other ocean worlds given that methanogens are deeply rooted in Earth’s tree of life and utilize a metabolism that could readily proceed independently of the input of sunlight energy. Given the ample supply of H_2_, methanogenesis is an energetically favorable process in serpentinizing systems and a growing body of evidence points to its occurrence therein (Miller et al., 2016; Canovas et al., 2017; Rempfert et al., 2017; Fones et al., 2021; Nothaft et al., 2021a; Nothaft et al., 2021b; Thieringer et al., 2023; Howells et al., 2022). However, serpentinization typically yields fluids that are highly depleted in dissolved inorganic carbon (DIC) and, at high pH, have exceedingly low concentrations of the methanogenic substrate CO_2_. The dissolved inorganic carbon concentration in pristine Type II serpentinized fluids can be as low as 20 μM (Howells et al., 2022). For the mesophilic, neutrophilic organism *Methanobacterium congolense*, Chen et al. (2019) report a Monod half-saturation constant for DIC of K_S-DIC_ = 2.2 mM, meaning that the cell will grow at half of its maximum rate at that DIC concentration. Below that concentration, the growth rate decreases approximately linearly with respect to DIC concentration. The implication of environmental DIC concentrations 100-fold lower than the reported K_S-DIC_ is, therefore, a 100-fold lower growth rate. The situation is compounded for CO_2_, which is indicated by experimental and X-ray crystallographic studies to be the form of DIC that reacts during the first step of methanogenesis (Vorholt and Thauer, 1997) in presently characterized methanogens: based on the pKa of carbonic acid (~6.5) and the pH of the fluids (~11.5) (see modeled CO_2_ concentrations in Howells et al., 2022) the difference in CO_2_ concentration is eight orders of magnitude. DIC availability may thus significantly limit methanogenesis in serpentinizing systems.

Previous work suggests that DIC availability may indeed be a substantial influence on methanogen ecology for fluids that have undergone the greatest extent of water-rock reaction (Type II). Fones et al. (2021) reported that methanogens of the genus *Methanobacterium*, associated with Type II fluids in the Samail ophiolite, have genomic potential for the uptake of formate and conversion of formate to CO_2_ for hydrogenotrophic methanogenesis (Fones et al., 2021). Furthermore, Type II *Methanobacterium* genomes show evidence for loss of H_2_ utilization, suggesting that those organisms may rely on formate as an electron donor instead. In parallel, incubations with ^14^C formate and ^14^C bicarbonate showed significant methanogenic activity with formate and no activity for bicarbonate in Type II fluids. In contrast *Methanobacterium* genomes from Type I fluids reflect an ability to carry out hydrogenotrophic methanogenesis with H_2_ as an electron donor, and methanogenesis from both formate and bicarbonate was observed. In agreement with Fones et al. (2021), Thieringer et al. (2023) observed the distinct Type I and Type II *Methanobacterium* genomes in shotgun metagenome sequencing data from recently drilled bore-hole fluids. However, in addition to formate utilization, they found genomic evidence for acetate uptake by *Methanobacterium* as a carbon source. No studies on serpentinized fluids in Oman have shown evidence for acetoclastic methanogens, namely of the families *Methanosarcinaceae* and *Methanosaetacea*. Potential acetoclastic methanogens of the family *Methanosarcinaceae* have only been detected in the seafloor hydrothermal serpentinized fluids of Lost City (McGonigle et al., 2020; Brazelton et al., 2022) and in no other serpentinizing systems studied to date (Colman et al., 2024).

The energy yield from acetoclastic and formatotrophic methanogenesis is generally lower than from CO_2_ reduction with H_2_ (Stams and de Sousa, 2019). However, these pathways could become more energetically favorable and support microbial life in environments where CO_2_ is limited but acetate or formate are available. Both acetate and formate can be produced abiotically in serpentinization-driven hydrothermal systems through Fischer-Tropsch-type (FTT) synthesis reactions, which involve the reduction of inorganic carbon (CO or CO_2_) with H_2_ under high-temperature, high-pressure conditions (McCollom and Seewald, 2007; McDermott et al., 2015) and therefore are a viable carbon source in serpentinizing systems. Both reactions, summarized in Table 1, can produce inorganic carbon, which can feed into the hydrogenotrophic pathways for methanogenesis. Furthermore, serpentinized fluids provide a unique opportunity to examine how low CO_2_ concentrations as a product of these reactions influence overall energy availability.

**Table 1:** Summary of redox reactions for methanogenic metabolisms examined in this study.

Methanogenic metabolism	Reaction
Hydrogenotrophic	4H_2_ + HCO_3_^−^ + H^+^ ➔ CH_4_ + 3H_2_O
Formatotrophic	4CHO_2_^−^ + H^+^ + H_2_O ➔ CH_4_ + 3HCO_3_^−^
Acetoclastic	C_2_H_3_O_2_^−^ + H_2_O ➔ CH_4_ + HCO_3_^−^

Multiple authors (Fones et al., 2021; Nothaft et al., 2021b; Thieringer et al., 2023; Colman et al., 2022) have pointed to low DIC availability as potentially influencing the microbial ecology of serpentinizing systems in Oman. In this study we quantitatively examine the influence of DIC availability on methanogenesis from three perspectives: (1) potential flux of substrates to the cell, (2) chemical affinity for the methanogenic metabolism, and (3) energy inventory per volume of fluid. Here, we focus on surface expressions of serpentinized fluids which allow ready access to significant chemical diversity (Canovas et al., 2017; Leong et al., 2021; Howells et al., 2022). Surface fluids largely retain both geochemical and biological similarity relative to subsurface fluids, while potentially also allowing us to consider the influence of mixing and atmospheric contact (Leong et al., 2021). For this study we collected and analyzed fluids for the concentration of acetate and formate and combined this new data set with DIC, H_2_ and CH_4_ values reported in Howells et al. (2022) for quantitative analyses of hydrogenotrophic, formatotrophic and acetoclastic methanogenesis (Table 1). We also performed shotgun metagenome sequencing on Type II fluid microbial communities to assess the genomic potential for these metabolisms. In carrying out a comparative energetic and genomic study, we broaden our understanding of possible metabolic strategies in extreme environments and inform our understanding of habitability.

## 2 Methods

### 2.1 Organic acid analysis

In this study, we characterized formate and acetate concentrations in surface-expressed fluids at sites, which are summarized in Table 2. The major ion fluid chemistry of these sites was previously reported on by Leong et al. (2021), and microbial community composition was previously reported by Howells et al. (2022) and Howells et al. (2023). Water samples from surface-expressed fluids were collected using a polytetrafluoroethylene (PTFE) scoop for organic acid analyses. The collected water was transferred to a one-liter Nalgene high-density polyethylene (HDPE) bottle. The bottle had a polypropylene tube inserted through a hole cut into the lid leading to a Covidien 140 mL plastic syringe. All plastic components were rinsed three times with each sample before filtration. This closed-system (albeit not airtight) setup was designed to minimize contamination and loss of volatiles from the sample as it was being apportioned into different types of sample containers.

**Table 2:** Formate and acetate data collected for this study.

Sample	Fluid location	Fluid	pH	Temp.	Si^a^	% Serp^b^	H_2_^a^	DIC^a^	Acetate	Formate
		Type		°C	Molality	Fluid	Molality	Molality	Molality	Molality
140115Z	Al Banah - Suface	Type II	11.3	32.2	1.4E-06	100.0	2.3E-04	3.4E-05	3.2E-06	3.0E-06
140115X	Al Banah - Surface	Type II	11.4	29.5	2.2E-06	99.9	2.3E-04	3.1E-05	4.1E-06	4.3E-06
140115Y	Al Banah - Surface	Type II	11.6	24.5	2.2E-06	99.9	2.1E-05	1.1E-04	2.9E-06	1.7E-06
140114S	Falej North - Surface	Mix	7.7	21	2.0E-04	76.5	1.5E-08	4.4E-03	8.5E-07	
140114U	Falej North - Surface	Type II	11.4	21.7	7.4E-06	99.3	1.7E-08	1.8E-04	2.5E-06	3.3E-06
140114V	Falej North - Surface	Type II	11.4	24.4	2.8E-06	99.8	1.7E-07	2.3E-04		9.8E-07
140114T	Falej North - Surface	Type II	11.4	27.2	1.8E-06	99.9	2.8E-05	4.2E-05	3.0E-06	5.5E-06
140114R	Falej North - Surface	Type II	11.6	21.1	2.4E-06	99.9	1.2E-08	4.2E-05		
140113O	Falej South - Surface	Type II	11.4	28.4	3.7E-06	99.7	3.9E-05	5.7E-05	3.9E-06	4.8E-06
140113P	Falej South - Surface	Type II	11.5	25.9	1.9E-06	99.9	6.6E-05	4.6E-05	2.2E-06	1.6E-06
140111G	Qafifah - Surface	Mix	8.9	22.6	2.8E-04	67.5	2.4E-07	4.1E-03	1.2E-06	
140111H	Qafifah - Surface	Mix	10.2	20.2	1.5E-04	83.0	1.1E-06	1.8E-03	6.6E-07	2.0E-06
140111I	Qafifah - Surface	Mix	10.9	18.8	1.0E-04	88.3	2.1E-06	8.5E-04	9.2E-07	1.3E-06
140111F	Qafifah - Surface	Type II	11.6	23.8	1.1E-05	98.9	2.6E-04	4.1E-05	1.8E-06	1.9E-06
140116B	Shmait - Surface	Mix	7.9	26.5	3.0E-04	64.4	6.2E-08	5.1E-03	1.5E-06	
140116C	Shmait - Surface	Mix	8.7	27.3	2.8E-04	67.2	2.2E-06	4.7E-03	1.6E-06	
140116D	Shmait - Surface	Mix	9.1	27.1	2.6E-04	69.4	1.3E-05	4.0E-03	1.4E-06	
140117J	Shmait - Surface	Mix	11.3	31.6	4.9E-05	94.4	2.2E-04	7.0E-05		5.3E-07
140117I	Shmait - Surface	Type II	11.3	32.3	2.0E-06	99.9	2.3E-04	4.7E-05		4.9E-07
140117G	Shmait - Surface	Type II	11.4	30.5	1.5E-06	100.0	2.8E-06	1.9E-05		1.5E-06
140117F	Shmait - Surface	Type II	11.5	26.2	2.7E-06	99.8	2.7E-04	2.9E-05	1.5E-06	1.2E-06
140117H	Shmait - Surface	Type II	11.5	29.6	1.7E-06	99.9	2.3E-04	2.9E-05		1.1E-06
140110B	Wadi Dima - Surface	Mix	8.4	23.5	1.8E-04	78.5	7.0E-09	3.6E-03		4.4E-07
140112L	Wadi Dima - Surface	Mix	9.8	21.3	1.1E-04	87.0	9.0E-09	1.9E-03		7.4E-07
140110D	Wadi Dima - Surface	Mix	10.4	21.8	9.1E-05	89.3	5.6E-08	8.1E-04		7.5E-07
140112M	Wadi Dima - Surface	Type II	11.4	28.2	5.6E-06	99.5	3.1E-05	5.2E-05		7.4E-07
140112K	Wadi Dima - Surface	Type II	11.4	26.9	3.9E-06	99.7	4.9E-07	3.6E-05	7.9E-07	9.4E-07
140110C	Wadi Dima - Surface	Type II	11.4	27	4.3E-06	99.6	6.4E-06	5.3E-05	1.3E-06	4.2E-06
NSHQ142014	Well*	Type II	11.4	34.9	7.0E-06	99.3	6.7E-04			
NSHQ142015	Well*	Type II	11.3	35.3	5.0E-06	99.6	2.9E-03	1.9E-04	1.2E-06	1.7E-06
NSHQ142016	Well*	Type II	11.2	35.8	5.0E-06	99.6	2.2E-04			
NSHQ212015	Well*	Mix	7.4	33.7	7.8E-04	7.8		2.6E-03	4.9E-07	1.1E-06
NSHQ3B2015	Well*	Mix	8.4	30.1	2.5E-04	71.0		2.5E-03	4.7E-07	1.2E-06
NSHQ42014	Well*	Mix	10.6	35.1	2.0E-04	76.8		2.2E-04	4.4E-06	1.5E-06
NSHQ42015	Well*	Mix	10.5	33.3	1.5E-05	98.4		1.8E-04	1.4E-06	2.3E-06
WAB1032015	Well*	Mix	8.2	30.4	4.8E-04	43.5		2.3E-03	4.3E-07	1.1E-06
WAB1032016	Well*	Type I	8.2	33.8	8.5E-04	0.0				
WAB1042016	Well*	Mix	8.5	33.4	2.1E-04	75.3				
WAB1052016	Well*	Mix	8.3	31.6	4.3E-05	95.1				
WAB1882015	Well*	Mix	8.7	34.2	2.3E-04	72.7		2.3E-03	3.8E-06	1.0E-06
WAB1882016	Well*	Mix	7.6	33	7.9E-04	7.4				
WAB552015	Well*	Type II	9.3	30	6.0E-06	99.4		2.6E-03	2.0E-06	1.4E-06
WAB552016	Well*	Type II	9.2	34.7	6.0E-06	99.4				
WAB562015	Well*	Type II	10.6	33.3	1.0E-05	99.0	1.8E-04			
WAB712015	Well*	Mix	11.0	33.1	1.7E-05	98.1			6.3E-07	1.5E-06
WAB712016	Well*	Mix	11.1	34.5	2.4E-05	97.3				

a Si, H_2_, and DIC values from Howells et al. (2022) (surface) and Rempfert et al. (2017) (well).

b % Serp. Fluid calculated from Si values in table and the modeled Type II fluid Si concentration reported in Howells et al. (2022).

* Formate and acetate values from Rempfert et al. (2017).

Water was then filtered through an Acrodisc^®^ Supor^®^ membrane 0.8/0.2 μm filter to remove particulates and cells. Prior to sample collection, >130 mL of sample was rinsed through the filters and not collected for the analyses. Approximately 20 mL of filtered water was then collected for organic acid anion analysis. Samples for organic acid analysis were collected in 20 mL Qorpak amber glass bottles with PTFE cap inserts. Prior to the field expedition, the amber glass bottles and their PTFE cap inserts were first rinsed three times with DI, and then the cap inserts were soaked for 24 h while the glass bottles were muffled in a furnace at 500°C for 24 h in order to remove any potential residual organic carbon. Then, the cap inserts were dried, and these bottles were also closed for transport to the field. Upon return from the field, the amber glass bottles were refrigerated until the time of analysis, which was under 5 weeks for all samples from the time of their collection.

In the lab, organic acid analysis was performed using a Dionex ICS-1500 ion chromatograph equipped with a Dionex IonPac^®^ ICE-AS6 ion exclusion column (9 × 250 mm), a Dionex AMMS-ICE 300 suppressor, a Dionex DS6 heated conductivity cell detector, and a Dionex AS40 autosampler. Samples were spiked with 25 μL of 3.75 M hydrochloric acid prior to analysis so that sample pH was <7 in order for organic acids to speciate into the proper proportions of associated versus dissociated for interaction with the column. Each sample was run twice, and each run consisted of duplicate injections. The first run used 0.60 mM heptafluorobutyric acid (HFBA) as an eluent and was designed to isolate the formate peak from eluting interferences. The second run used 0.15 mM HFBA to isolate the acetate peak. In both cases, the eluent flow rate was set to 1.00 mL/min. The suppressor regenerant was 5.00 mM tetrabutylammonium hydroxide (TBAOH), set to a nitrogen pressure-driven flow of ~3 mL/min. Five-point calibration curves with R^2^ > 0.993 were generated for formate and acetate using standards purchased from High-Purity Standards. Natural sample peaks were verified in all cases by overlaying sample and standard chromatograms and, in some cases, verified by standard addition. Instrument detection limits for organic acid anions were ~0.2 μM.

### 2.2 Energetic calculations

The concentration of major ions and the concentration of Si in this study were previously reported on by Leong et al. (2021) and Howells et al. (2022). For details on methods for fluid sample collection and analyses, please see those studies. Chemical speciation of site fluids to determine chemical activities of reactants and products for methanogenesis reactions summarized in Table 1 were performed on the Water-Organic-Rock-Microbe (WORM) Portal^1^ using the AqEquil Python package (Boyer et al., 2024) and wrapper for EQ3/6 (Wolery, 2013). During speciation, all redox reactions involving N, C, S, and P were suppressed. The charge balance is set based on pH or Cl^−^ (or no charge balance), depending on the data available for each sample site. Redox state was set based on O_2_ for fluids with greater influence from the atmosphere (Type I and mix) and H_2_ for pristine serpentinized fluid (Type II). Chemical affinities and energy supplies for redox reactions were calculated for speciated samples using the calculate_energy function included in AqEquil. This function calculates the chemical affinity of a given reaction, *r*, using the equation published in Shock et al. (2010),


(1) $$A_{r}\text{≡}-{\left(\frac{\delta \Delta_{r}G}{\delta \xi_{r}}\right)}_{P,T}$$

which is Gibbs energy change of reaction, *r*, (Δ*_r_G*) with respect to reaction progress (ξ_r_). Δ*_r_G* is solved by,


(2) $$\Delta_{r}G=\Delta_{r}G^{{}^{\circ}}+\mathrm{RTln}Q_{r}$$

with *R* being the universal gas constant, *T* the temperature of the fluid in Kelvin, Δ*_r_G°* the standard state Gibbs energy of the reaction given by


(3) $$\Delta_{r}G^{{}^{\circ}}=-\mathrm{RTln}K_{r}$$

and *Q_r_* the activity product given by


(4) $$Q_{r}=\prod {\left(a_{i}\right)}^{v_{i,r}}$$

*Q_r_* is solved with the chemical activities estimated using the AqEquil package described above where *a_i_* is the activity (*a*) of the given reactant or product (*i*) raised to its stoichiometry in the reaction (*v_i,r_*) (positive for products and negative for reactants). From Equations 1–4 we can solve for affinity,


(5) $$A_{r}=\mathrm{RTln}\left(\frac{K_{r}}{Q_{r}}\right)$$

Chemical affinities calculated using Equation 5 were normalized according to the number of moles CH_4_ produced (kJ per mole CH_4_). Energy supplies for each reaction (*E_r_*) (J per kg fluid) were calculated using equations outlined in Howells et al. (2022). Briefly, affinity (*A_r_*) in J per mole reaction is multiplied by the concentration of the limiting reactant (*m_lim_*) and divided by the stoichiometric coefficient of the limiting reactant (*v_lim_*), as summarized below.


(6) $$E_{r}=\frac{A_{r}\left[m_{\lim}\right]}{v_{\lim}}$$

Using Equation 6, energy supplies were calculated in AqEquil with the parameter as_written set to False to sum all chemical species in equilibrium with a designated substrate. This accounts for the fact that as the substrate is consumed in 1 kg of fluid, the system re-equilibrates to supply more of that substrate. For example, as HCO_3_^−^ is consumed from a pool of inorganic carbon, the system re-equilibrates with other inorganic carbon species (CO_2(aq)_, CO_3_^2−^, CaHCO_3_^+^, etc.) to continuously supply CO_2_ until the whole inorganic carbon pool is consumed. The other option in AqEquil is to set the parameter as_written to True, which only uses the concentration of the substrate specified in the reaction and no other (e.g., HCO_3_^−^ and no other forms of DIC), however this option was not used in the current study. The output of AqEquil with all estimated chemical activities can be found in Supplementary Table S1.

### 2.3 Meta-analysis of 16S rRNA gene amplicon sequencing

For the meta-analysis of microbial diversity in subsurface well and surface-expressed serpentinized fluids we downloaded raw 16S rRNA gene amplicon sequencing data from NCBI Short Read Archive (SRA). For well fluids, we downloaded data from SRA accession SRP092764 (Rempfert et al., 2017). For surface data we downloaded from SRA accession, SRP308538 (Howells et al., 2022). Both sets of data were generated using the EMP primer set (Caporaso et al., 2012) and Illumina MiSeq sequencing platform. Rempfert et al. (2017) used the 2 × 250 Illumina method, Howells et al. (2022) used the 1 × 150 Illumina method. Raw data downloaded from NCBI SRA was processed using programs within the QIIME2 version 2021-4 wrapper following protocol described in Howells et al. (2022). Briefly, sequencing quality was checked using FastQC and trimmed based on a quality score of 25 or lower. Sequences were denoised and resolved to 100% sequence similarity with DADA2 (Callahan et al., 2016). The resulting amplicon sequence variants (ASVs) were rarefied to 2,500 counts per sample. Alpha diversity analysis carried out in QIIME2 using the q2-diversity plugin. Taxonomic assignment was done using the QIIME2 plugin, q2-feature-classifier with the naïve Bayes method with pre-trained classifier built using the Green Genes 13_8 99 16S rRNA gene database (DeSantis et al., 2006). We acknowledge Green Genes is an older database, however we find that Green Genes produces comparable taxonomic assignments as the more recent SILVA ribosomal RNA gene database (Quast et al., 2012) as shown by Howells et al. (2023). The rarefied ASV table was exported from QIIME2 and beta diversity (NMDS) analysis carried out with the R package, vegan (version 2.5-6) within the R version 3.6.0 (2019-04-26) (R Core Team, 2021). For the NMDS analysis, rarefied frequencies of ASVs at each site were converted to relative abundances and square-root transformed. Bray–Curtis dissimilarity was calculated on the transformed relative abundances. NMDS ordination was conducted with 1,000 permutations and two dimensions. The stress for the NMDS ordination was 0.2. Supplementary Table S2 is the relative abundance of each ASV at each study site and is used for downstream statistical analyses. Supplementary Table S3 relative abundance of ASVs grouped at the genus level (taxonomy included).

### 2.4 Shotgun metagenome sequencing

Sediments for shotgun metagenome sequencing were collected simultaneously for 16S rRNA gene amplicon sequencing using the same approach described in Howells et al. (2022) from sites 140112K, 140117H, and 140111F. Pictures of the sampling sites and a description of the sediments can be found in Supplementary Figure S1. In the lab, DNA was extracted using the Quick-DNA Miniprep Plus Kit (catalog #D4068) from Zymo. For each Zymo prep, we extracted DNA from ~500 mg (kit protocol recommends 200 mg, but 500 mg was optimal for our sediments) of wet sediment in replicate for each site and pooled the DNA. For site 140112K, 8 replicate DNA extracts were pooled; for site 140117H, 8 replicates were pooled; and for 140111F, 5 replicates were pooled. We implemented the Zymo protocol for “BioFluid + Cell Buffer.” Prior to carrying out the extraction protocol provided by Zymo, we conducted freeze/thaw cycles where samples were first thawed at room temperature, then frozen at −80°C for 15 min, heated to 55°C for 15 min three times. We carried out a proteinase K step, adding 10 uL of 20 μg/mL proteinase K per 100 mg of wet sediment and incubated at 55°C for 3 h. From there, the “BioFluid + Cell Buffer” protocol was followed. The quality of the DNA extracts was assessed using a NanoDrop. The A260/A280 of the extracts were between 1.5 and 2. PCR was conducted to ensure extracts were PCR amplifiable. DNA extracts were then pooled. The sequencing facility was provided 35 ng DNA from site 140112K, 30 ng DNA from site 140117H, and 60 ng from site 140111F. Shotgun metagenome sequencing was conducted in the Marine Biological Laboratory at Woods Hole Oceanographic Institute using the Illumina 150 × 2 platform using their standard protocol.

We processed shotgun metagenome sequencing reads in two ways, “by-site” and “co-assembly.” For our by-site approach, we processed sequencing data from each site individually. Raw sequencing reads, both forward and reverse, in “.fastq” format were loaded into The Department of Energy Systems Biology Knowledgebase (KBase).^3^ In Kbase, the quality of raw sequencing reads was assessed using FastQC v0.12.1.^4^ Based on the quality assessment, files were trimmed with the program Trimmomatic v0.36 (Bolger et al., 2014) with a “sliding window size” of 4 and a “sliding window minimum quality” of 25. Once the read files were trimmed, their quality was rechecked using FastQC v0.12.1. Once the quality of the reads was determined to be sufficient, the reads were assembled using metaSPAdes v3.15.3 (Nurk et al., 2017). In MetaSPAdes, the “Minimum Contig Length” parameter was less than or equal to 1,000, with the smallest possible read length being 300. MetaSPAdes produced 32,933 contigs from site 140112K, 68,329 contigs from site 140117H, and 60,294 contigs from site 140111F. The contigs produced were annotated with RASTtk v1.073 using the default KBase parameters.

Contigs were binned using CONCOCT v1.1 (Alneberg et al., 2013) and MetaBAT2 v1.7 (Kang et al., 2019), with the maximum contig length parameter being less than or equal to 2,500 base pairs. The lowest possible contig length for CONCOCT and MetaBAT2 was set to 1,500 base pairs. CONCOCT binned 17,914 contigs from site 140112K, 36,751 contigs from site 140117H, and 41,353 contigs from site 140111F to produce 62, 84, and 87 bins from each site, respectively. MetaBAT2 binned 13,460 contigs from site 140112K, 28,028 contigs from site 140117H, and 32,614 contigs from site 140111F to produce 40, 60, and 49 bins from each site, respectively. We optimized the bin outputs from CONCOCT and MetaBAT2 with DAS Tool v1.1.2 (Sieber et al., 2018) using the default diamond identification tool and default advanced parameters. The bin optimization tool resulted in 22 optimized bins from site 140112K, 41 bins from 140117H, and 36 bins from 140111F.

The quality of the bins produced was assessed with CheckM v1.0.18 (Parks et al., 2015). Following bin assessment, all the bins were extracted as assemblies using Extract Bins as Assemblies from BinnedContigs v1.0.2 in Kbase. Once the bins were extracted, the taxonomy of each bin was classified using GTDB-tk v1.7.0 (Chaumeil et al., 2022), and bins classified as known methanogens were functionally annotated using RASTtk v1.073 (Brettin et al., 2015) using the default KBase parameters.

All quality-checked and trimmed “.fastqc” sequencing files were uploaded into one read file using the program Merge Reads Libraries v1.2.2 in Kbase for the co-assembly approach. From there, contigs were assembled using metaSPAdes. This resulted in 121,536 contigs. Contigs were then binned following the same protocol as the by-site approach. The number of bins resulting from CONCOCT v1.1 was 135 from 121,536 contigs, and the number of bins from MetaBAT2 v1.7 was 96 from 56,113 contigs. Bin optimization was carried out using DAS Tool v1.1.2, resulting in 64 bins. Assemblies were extracted and taxonomically classified using GTDB-tk v1.7.0. Those classified as methanogens were functionally annotated using RASTtk v1.073.

The relative frequency of each bin at each site was determined using the program Bowtie2 v2.3.2 (Langmead and Salzberg, 2012) in Kbase with phred33 used as the alignment quality score type, the alignment type set to end-to-end and the maximum fragment length for paired-end alignments set to 500. Bowtie2 maps raw sequence reads against genome assemblies to determine the percentage of reads that align with each assembly at each site.

After assembling and processing the bins, we searched the RASTk annotations for core methanogen metabolism genes using Thieringer et al. (2023) as a guide. We used BLASTp (Camacho et al., 2009) to determine the percent identity with protein sequences of previously characterized methanogen genomes. Supplementary Table S4 contains tables of RASTtk annotations and BLASTp results of amino acid sequences that code for proteins in the core metabolisms of the resolved methanogen genomes.

### 2.5 Phylogenomic analysis

GToTree was used to construct a phylogenomic tree of methanogens (Lee, 2019). We included several genomes from the Samail Ophiolite isolated by Thieringer et al. (2023), Fones et al. (2019), and those newly published in this study. We also curated and included several high-quality genomes for which environmental metadata was available. Additionally, representative genomes from the genera *Methanobacterium*, *Methanosphaera*, *Methanobrevibacter*, and *Methanosarcina* were included using the “gtt-get-accessions-from-GTDB” functionality with the “--GTDB-representatives-only” flag for each of these taxa. Finally, genomes identified by GTDB-Tk - v2.3.2 as “close relatives” of *Methanocalculus natronophilus*, *Methanobacterium congolense* buetzberg, and the Oman MAGs isolated by Thieringer et al. (2023) and Fones et al. (2019) were also included. However, for genomes identified by GTDB, we removed those that were shown to be redundant in previous maximum parsimony and maximum likelihood trees from the final phylogeny for the sake of clarity. A full table of the genomes used and any associated metadata used in this study is available in Supplementary Table S5.

Once the genomes were collected, GToTree v1.8.8 was run with default parameters, using the hidden Markov model (HMM) target gene set of 76 archaeal marker genes included in the program as the basis for the phylogeny. Genes were predicted within input genomes using Prodigal v2.6.3 (Hyatt et al., 2010); our 76 target genes were then identified within these genes using HMMR v3.4 (Eddy, 2011) and, respectively, aligned using Muscle 5.1 for OS x 64 (Edgar, 2022). These genes were then trimmed via TrimAl v1.5.rev0 (Capella-Gutiérrez et al., 2009) and concatenated to construct the phylogenomic tree. Then, IQ-TREE v2.3.6 (Nguyen et al., 2015) was used to generate a maximum-likelihood phylogeny with 1,000 bootstrap replicates. Subsequently, the tree was visualized in the Interactive Tree of Life online viewer (Letunic and Bork, 2024). Canva was then used to overlay relevant environmental metadata onto the tree and reformat taxon names. Supplementary Table S5 includes optimal growth temperature and pH for genomes of methanogens in culture and the temperature and pH of sampling sites of metagenome-assembled genomes in this study and other studies where relevant.

## 3 Results and discussion

### 3.1 Surface-expressed serpentinized fluids, a window into subsurface processes

**Figure 1:**
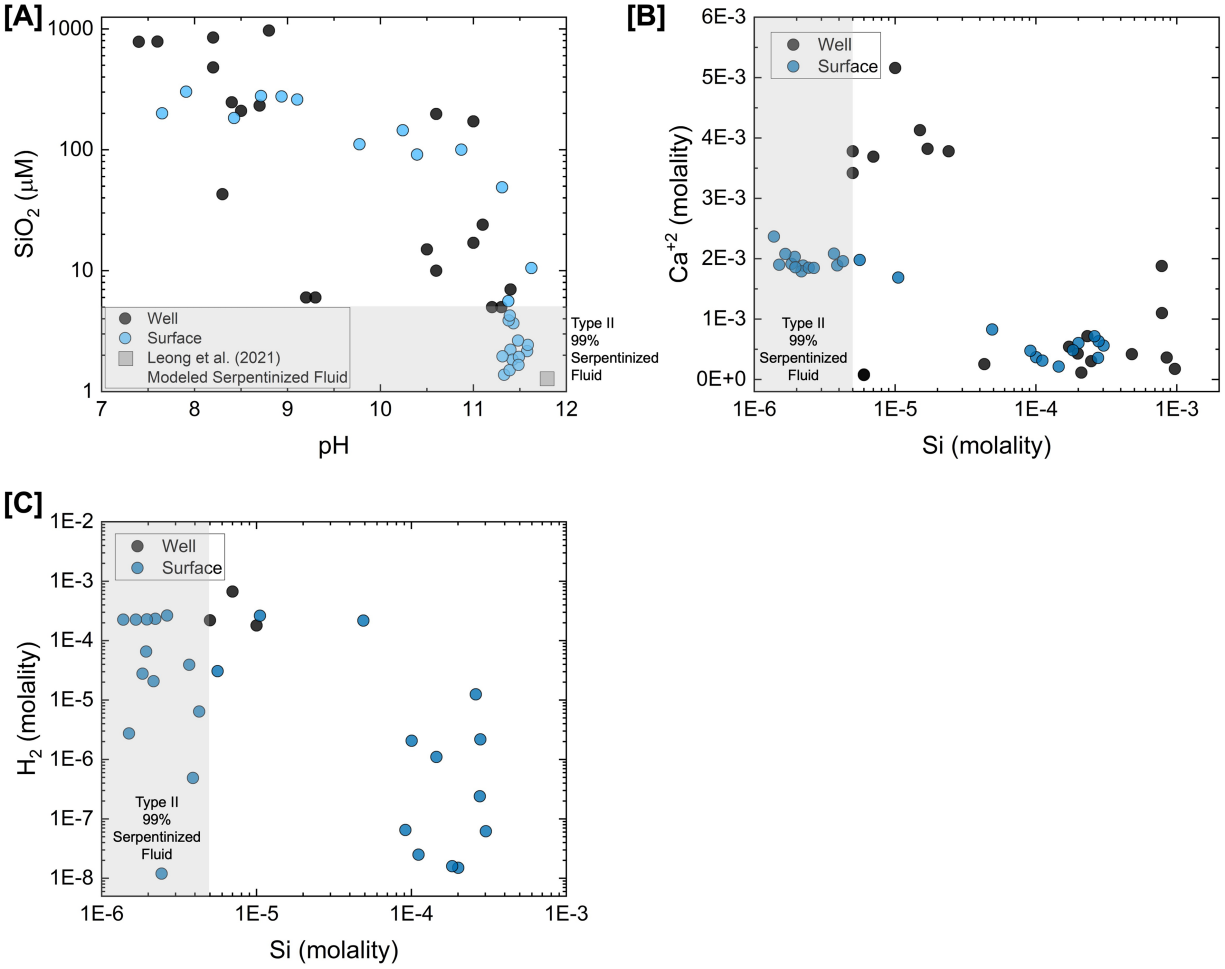
Comparison of surface-expressed and subsurface well serpentinized fluid chemistry from the Samail Ophiolite. Chemical constituents plotted are directly influenced by the process of serpentinization as described in Leong et al. (2021). Values for surface-expressed fluid chemistry (blue) are reported in Howells et al. (2022) values from well fluids (black) are reported in Rempfert et al. (2017). This figure is adapted from Howells et al. (2022). **(A)** The concentration of Si in fluids plotted as a function of pH. Si is considered to be an indicator of the extent of serpentinization fluids have undergone and extent Type I and Type II fluids have mixed (Leong et al., 2021). The gray square represents the estimated pH and Si concentration of a pristine serpentinized fluid modeled by Leong et al. (2021). This is used as the Type II end-member fluid for estimating the percent serpentinized fluid composition of fluids using a conservative mixing model with Si as described in Howells et al. (2022). **(B)** Concentration of Ca^+2^ plotted as a function of Si. **(C)** Concentration of H_2_ plotted as a function of Si.

To illustrate geochemical similarities and differences between surface-expressed and subsurface well fluids, Figure 1 summarizes the concentrations of geochemical constituents reported in Howells et al. (2022) and Rempfert et al. (2017) directly influenced by the process of serpentinization. Leong et al. (2021) describe how the serpentinization process influences pH, Si, Ca, and DIC in detail. Si indicates the extent to which fluids have undergone serpentinization and the extent of mixing between Type I (high Si concentration) and Type II (low Si concentration) fluids. Based on the concentration of Si and pH shown in Figure 1A, there is a high degree of geochemical overlap between surface and subsurface fluids. However, Figure 1B shows a distinction in the concentration of Ca, with more pristine Type II fluids (lowest concentrations of Si) from the subsurface having higher concentrations of Ca. This may be attributed to Ca in surface fluids reacting with inorganic carbon supplied from CO_2_ in the atmosphere to form carbonate minerals, which can draw down the Ca concentration in serpentinized fluids. Additionally, in fluids where H_2_ was measurable in the Rempfert et al. (2017) study, H_2_ is more than an order magnitude higher in concentration than the highest H_2_ concentrations from the surface, as shown in Figure 1C. Lower H_2_ concentrations in surface fluids may be attributed to the consumption of H_2_ by microorganisms or degassing of H_2_ from serpentinized fluid along its migration from the subsurface to the surface. Despite these differences, previous energetic calculations show sufficient energy for hydrogenotrophic methanogenesis in more pristine Type II surface-expressed fluids (Canovas et al., 2017; Howells et al., 2022). Energetic observations coupled with the detection of methanogen 16S rRNA gene phylotypes in Howells et al. (2022) suggest that H_2_ would not be a limiting factor in many surface-expressed fluids and certainly not in subsurface fluids. The difference in Ca concentration provides insights into factors that may influence inorganic carbon availability in the surface and subsurface.

**Figure 2:**
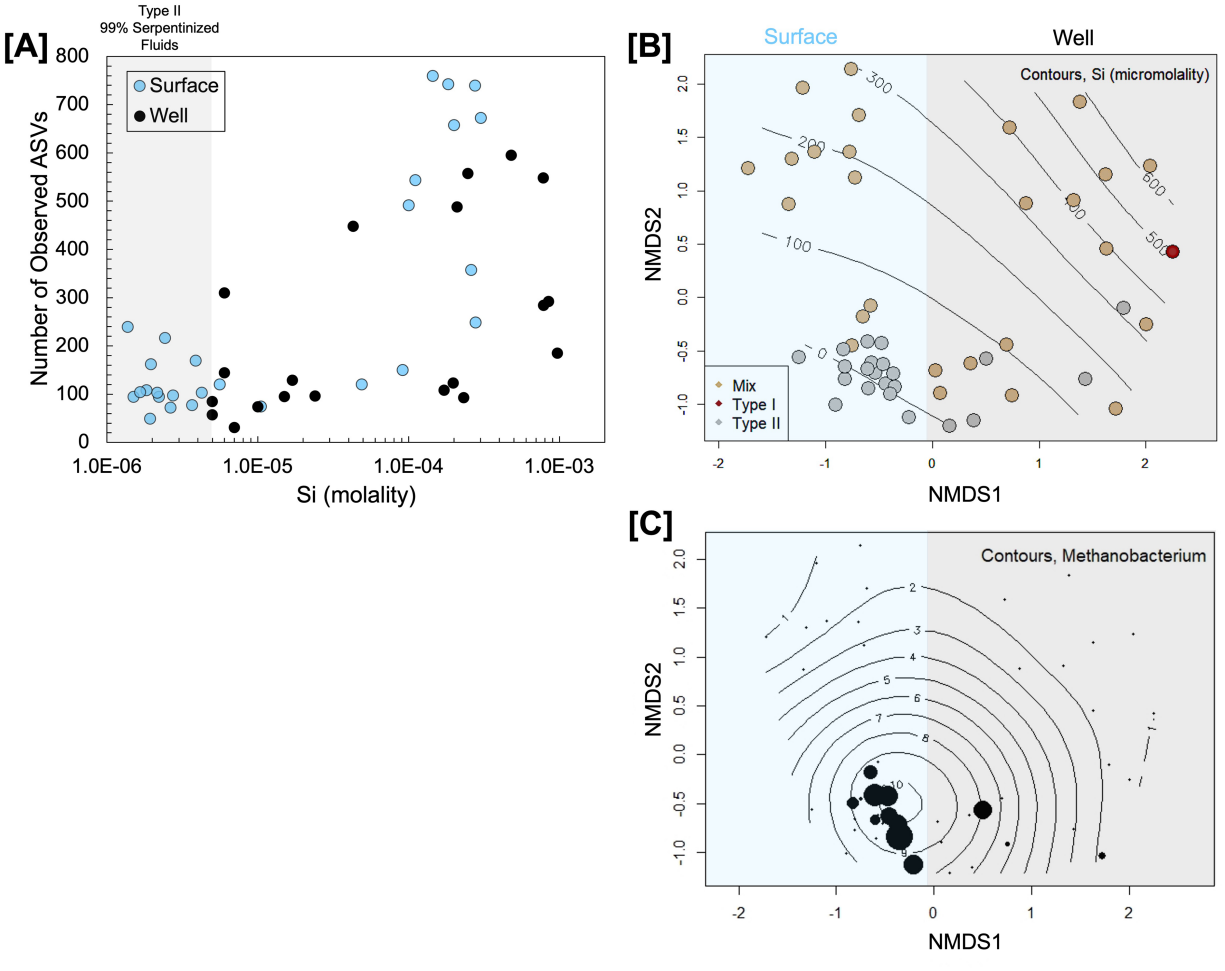
Comparison of microbial community diversity in surface-expressed and subsurface well serpentinized fluids of the Samail Ophiolite. 16S rRNA gene amplicon sequencing data from Rempfert et al. (2017) and Howells et al. (2022) were reprocessed to compare the alpha diversity quantified through the number of observed ASVs plotted as a function of Si in **(A)** and the beta diversity assessed through Bray–Curtis dissimilarity analysis of square-root transformed ASV relative abundances visualized through NMDS ordination (stress = 0.2) in **(B)**. Contours in **(B)** are a maximum likelihood fit of the Si concentration at each site and colors indicate fluid type following Table 2. The distribution of 16S rRNA gene amplicon ASVs classified as *Methanobacterium* is shown by the size of circles in **(C)**, which scales with relative abundance and. Contours in **(C)** are a maximum likelihood fit of the relative abundance of *Methanobacterium*.

To examine similarities in microbial community composition and the distribution of *Methanobacterium* in surface-expressed and subsurface well fluids, we conducted a meta-analysis of 16S rRNA gene amplicon sequencing data reported in Howells et al. (2022) (surface-expressed fluids) and Rempfert et al. (2017) (subsurface well fluids). We can do this meta-analysis given that both studies used the Earth Microbiome Primer set (Caporaso et al., 2012) and the same type of sequencing platform (MiSeq 150 × 2). In comparing the alpha diversity, or the number of distinct representative 16S rRNA gene sequences, of surface and subsurface communities, we see in Figure 2A that both surface and subsurface fluid alpha diversity negatively trend with Si. It is worth noting that surface-expressed fluids can have higher alpha diversity than subsurface fluids with <99% serpentinized fluid composition. Both subsurface and surface-expressed Type II fluids have relatively low alpha diversity in comparison to Type I and mixed fluids, which in previous studies is hypothesized to result from the selective pressure of alkaline pH (Rempfert et al., 2017; Fones et al., 2019; Fones et al., 2021).

To assess beta diversity or similarities in community composition, we carried out an NMDS ordination of a Bray–Curtis dissimilarity analysis of square-root transformed 16S rRNA gene ASV relative abundances. Visually, community composition variation along NMDS1 trends with whether the communities are from the surface or the subsurface, as shown in Figure 2B. ANOSIM analysis shows that the communities from the surface and subsurface are significantly different (ANOSIM r statistic = 0.5124, *p*-value = 0.0001, 9,999 permutations); however, Type II fluids from the surface and subsurface fall closer together in ordination than fluids influenced by mixing and Type I fluids. Mixing and Type I fluids are likely dissimilar between the surface and subsurface due to the availability of O_2_ and sunlight at the surface. As Howells et al. (2023) reported, surface fluids influenced by mixing are populated by phototrophs and heterotrophs that use O_2_ as an electron acceptor. The dissimilarity between the Type II fluid communities may be due to the differences in the relative abundance of *Methanobacterium* and OP1 [also referred to as Acetothermia (Colman et al., 2022)] as shown by the NMDS ordination with OP1 relative abundances overlaid in Supplementary Figure S2. We overlayed the relative abundance of *Methanobacterium* phylotypes onto the NMDS ordination, as shown in Figure 2C. *Methanobacterium* is present in both Type II fluid types; however, it appears to be slightly more prevalent in surface-expressed Type II fluids. Overall, the community analysis shows that while there are significant global differences between surface and subsurface fluids, as may be expected due to differences in the availability of O_2_ and sunlight, Type II fluids are surprisingly similar in alpha diversity and community composition. Furthermore, *Methanobacterium* is prevalent in surface-expressed and subsurface Type II fluids.

### 3.2 Energetic potential for methanogenesis

**Figure 3:**
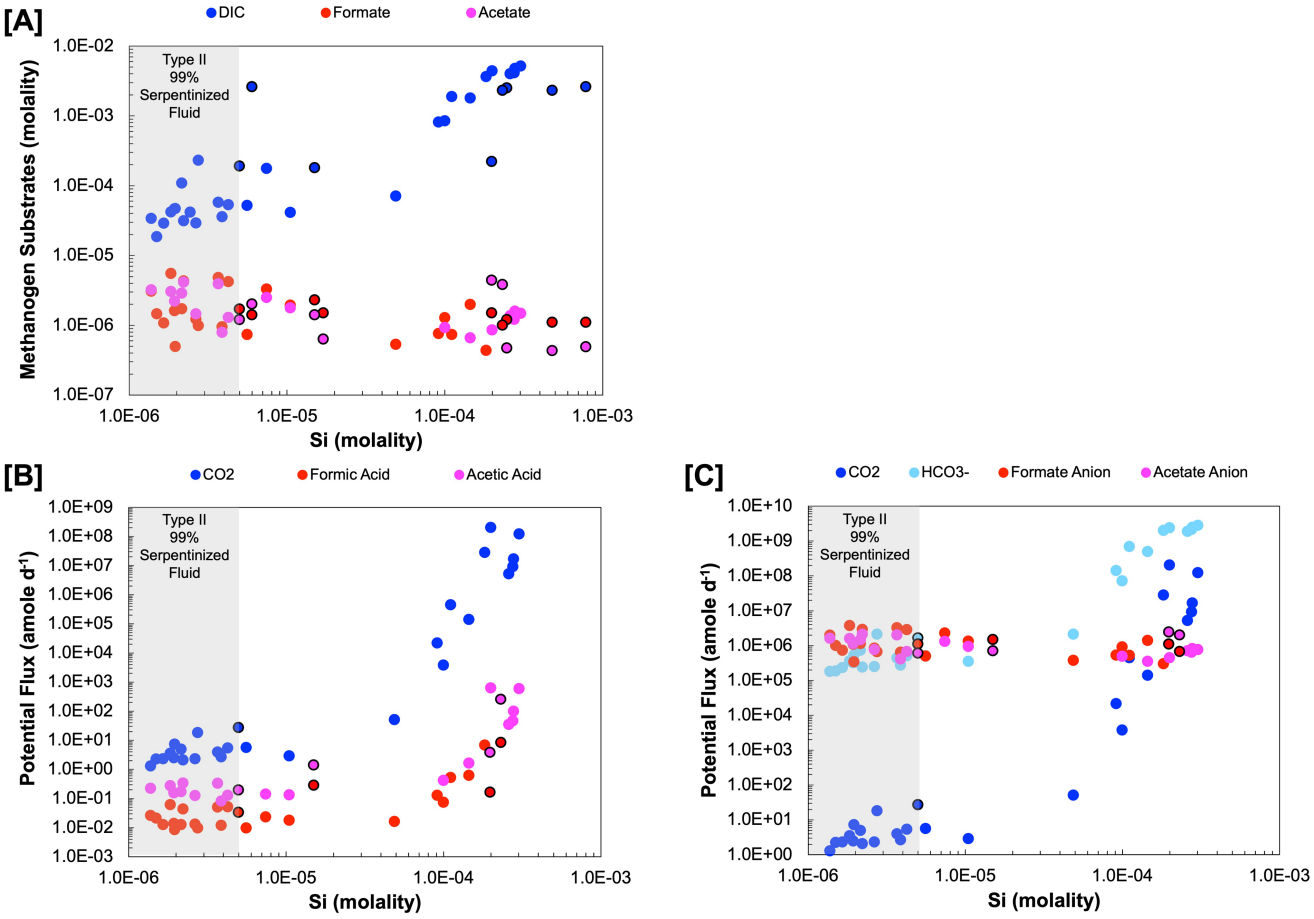
Substrate concentration in molality and potential flux in attomoles per day (*a*mole day^−1^) **(A)** Are the measured concentrations of dissolved inorganic carbon, formate and acetate. **(B)** is the potential flux for the acidic forms, dissolved CO_2_ (carbonic acid), formic acid, and acetic acid. **(C)** is the approximation of potential flux for the anion forms HCO_3_^−^, formate anion and acetate anion. Black outlines indicate subsurface well fluids.

In this section we quantitatively examine methanogen substrate availability through assessments of substrate flux to the cell, availability of energy in terms of moles CH_4_ produced and energy supply per kg of fluid. For these assessments we characterized formate and acetate concentrations shown in Figure 3A in surface-expressed fluids and combine our data with DIC, H_2_ and CH_4_ concentrations previously reported in Leong et al. (2021) and Howells et al. (2022), which are from the same sites and sampled at the same time. Some well fluids characterized in Rempfert et al. (2017) have measured concentrations of H_2_, CH_4_, DIC, acetate, and formate allowing comparison between surface and subsurface. Table 2 summarizes the organic acid data collected for this study as well as organic acid data previously reported in Rempfert et al. (2017). Also in Table 2 is an estimation of the percent serpentinized fluid at each study site using the concentration of Si as described in Howells et al. (2022) and a descriptor of the sampling site as Type II (>99% serpentinized fluid), mixed, and our representative Type I surface expressed fluid. As described in the methods, we estimate the chemical activities of dissolved CO_2_ and HCO_3_^−^ from measurements of DIC. We account for the effects of major ion concentrations (such as Na^+^, Cl^−^, Ca^+2^ and Mg^+2^) and pH on the chemical speciation of DIC and activity of dissolved CO_2_ and HCO_3_^−^ using the program EQ3/6 (Wolery, 2013) and program wrapper we developed, AqEquil Python package (Boyer et al., 2024). Given the alkalinity of serpentinized fluids we cannot measure CO_2_ through the gas phase as we did with H_2_ and CH_4_ in Howells et al. (2022). Therefore, estimating dissolved CO_2_ in this manner is the best approach for deriving a CO_2_ chemical activity. The same approach was used to estimate the chemical activity of formic acid, formate anion, acetic acid and acetate anion from bulk measurements of formate and acetate, respectively. Estimated chemical activities can be found in Supplementary Table S1. We assume chemical activities are approximately equal to molarity for the potential flux calculations described below.

#### 3.2.1 Potential flux

Low concentrations can limit the diffusive flux of substrates to a cell and thereby limit rates of metabolism. We calculated the potential fluxes of methanogenic substrates as follows. The rate of diffusive flux to the surface of a sphere of radius R is given by integrating the general equation relating flux and substrate concentration in spherical coordinates (Crank, 1979) from R to infinity (Hoehler, 2004):


(7) $$J=4\pi DR\cdot \left(C_{\infty }-C_{R}\right)$$

Where *J* is the diffusive flux, *D* is the species-specific diffusion coefficient, *C*_∞_ is the substrate concentration at infinite distance from the cell, and *C_R_* is the substrate concentration at the surface of the sphere. In our calculations, *R* is set to 0.5 μm in order to yield the flux to a spherical surface of cell-like scale. *C*_∞_ is set to the measured bulk solution concentration of a given substrate. In practice, “infinite distance” with respect to a cell consuming substrate at the diffusion limited rate is on the order of tens of microns (Boone et al., 1989), meaning that the substrate concentration at that distance very closely approximates the substrate concentration at true infinite distance. C_R_ is set to the concentration of substrate that is at equilibrium with respect to the methanogenic metabolism in question, in order to place a lower bound on the possible extent of substrate depletion at the surface of the sphere that could result from metabolic consumption. In practice, C_R_ would almost certainly be higher because (i) cells can only consume substrates down to concentrations that still support energy conservation, rather than to equilibrium; and (ii) enzymatic substrate consumption at less than the diffusion-limited rate implies that substrate concentrations at the surface of the sphere will be drawn down to only a limited extent. The latter of these considerations is particularly important because most enzymes operate orders of magnitude more slowly than the diffusion-limited rate (Bar-even et al., 2011), and the potential draw down of substrate concentration at R will be limited to the same extent. For these reasons, the calculated fluxes represent physical upper limits on the possible rates of substrate delivery to a cell, and we note this by henceforth referring to them as “potential fluxes.”

Separate calculations were performed for potential fluxes of the neutral species CO_2_, formic acid, acetic acid, which can potentially diffuse freely across a cell membrane, and the corresponding anionic forms, HCO_3_^−^, formate, and acetate, which can only transit the cell membrane via facilitated diffusion (e.g., through ion channels) or active transport. The calculated fluxes are specific to a given compound and neglect the potential for resupply by equilibration with protonated or ionic counterparts. For example, calculated fluxes assume that dissolved CO_2_ is delivered to the cell surface solely by diffusion from the bulk medium and not via production from bicarbonate in the vicinity of the cell surface. At the length scales considered here, diffusion dominates over the comparatively sluggish kinetics of CO_2_ production from bicarbonate (Johnson, 1982), making this approach a good approximation for the DIC system. It is not clear whether the same is true for the potential for formic acid and acetic acid to be formed in the vicinity of the cell by protonation of formate and acetate, respectively, and the potential fluxes of formic and acetic acids could be higher than the calculated values as a consequence. Table 3 shows the diffusion coefficients (*D*) used, their references, and the potential fluxes calculated using Equation 7.

**Table 3:** Potential flux values reported in attomoles per day (*a*mole d^−1^) and the diffusion coefficients in water (*D*) at 25°C, 1 bar for each chemical species used in the calculation.

Sample	SiO_2_	CO_2_	Formic acid	Acetic acid	HCO_3_^−^	Formate anion	Acetate anion
	Molality	*a*mole d^−1^	*a*mole d^−1^	*a*mole d^−1^	*a*mole d^−1^	*a*mole d^−1^	*a*mole d^−1^
140115Z	1.4E-06	1.3E+00	2.6E-02	2.2E-01	1.8E+05	2.0E+06	1.6E+06
140115X	2.2E-06	2.1E+00	4.3E-02	3.3E-01	2.4E+05	2.9E+06	2.1E+06
140115Y	2.2E-06	4.9E+00	1.3E-02	1.7E-01	7.3E+05	1.1E+06	1.5E+06
140114S	2.0E-04	2.0E+08		6.2E+02	2.4E+09		4.4E+05
140114U	7.4E-06		2.3E-02	1.4E-01		2.3E+06	1.3E+06
140114V	2.8E-06	1.8E+01	9.5E-03		2.1E+06	6.6E+05	
140114T	1.8E-06	3.5E+00	6.1E-02	2.7E-01	3.6E+05	3.7E+06	1.6E+06
140114R	2.4E-06						
140113O	3.7E-06	3.9E+00	5.0E-02	3.3E-01	4.3E+05	3.2E+06	2.0E+06
140113P	1.9E-06	2.4E+00	1.4E-02	1.5E-01	3.2E+05	1.1E+06	1.1E+06
140111G	2.8E-04	9.1E+06		4.6E+01	2.1E+09		6.3E+05
140111H	1.5E-04	1.4E+05	6.1E-01	1.6E+00	4.9E+08	1.4E+06	3.5E+05
140111I	1.0E-04	3.8E+03	7.3E-02	4.1E-01	7.1E+07	9.0E+05	4.9E+05
140111F	1.1E-05	2.9E+00	1.8E-02	1.3E-01	3.5E+05	1.3E+06	9.3E+05
140116B	3.0E-04	1.2E+08		5.8E+02	2.8E+09		7.5E+05
140116C	2.8E-04	1.7E+07		9.8E+01	2.5E+09		8.1E+05
140116D	2.6E-04	5.2E+06		3.5E+01	1.9E+09		7.0E+05
140117J	4.9E-05	5.1E+01	1.6E-02		2.1E+06	3.7E+05	
140117I	2.0E-06	7.2E+00	8.3E-03		5.2E+05	3.4E+05	
140117G	1.5E-06	2.2E+00	2.1E-02		1.9E+05	9.9E+05	
140117F	2.7E-06	2.3E+00	1.3E-02	1.2E-01	2.5E+05	8.3E+05	7.6E+05
140117H	1.7E-06	2.3E+00	1.3E-02		2.3E+05	7.3E+05	
140110B	1.8E-04	2.8E+07	6.7E+00		2.0E+09	3.0E+05	
140112L	1.1E-04	4.4E+05	5.2E-01		6.9E+08	5.2E+05	
140110D	9.1E-05	2.1E+04	1.3E-01		1.4E+08	5.3E+05	
140112M	5.6E-06	5.6E+00	9.6E-03		5.0E+05	5.0E+05	
140112K	3.9E-06	2.7E+00	1.2E-02	8.0E-02	2.7E+05	6.4E+05	4.1E+05
140110C	4.3E-06	5.3E+00	5.1E-02	1.3E-01	5.0E+05	2.8E+06	6.7E+05
NSHQ212015*	7.8E-04						
WAB1882016*	7.9E-04						
WAB1032015*	4.8E-04						
WAB1032016*	8.5E-04						
WAB1052016*	4.3E-05						
NSHQ3B2015*	2.5E-04						
WAB1042016*	2.1E-04						
WAB1882015*	2.3E-04		8.2E+00	2.5E+02		6.7E+05	2.0E+06
WAB552016*	6.0E-06						
WAB552015*	6.0E-06						
NSHQ42015*	1.5E-05		2.8E-01	1.4E+00		1.5E+06	7.0E+05
NSHQ42014*	2.0E-04		1.6E-01	3.8E+00		1.1E+06	2.4E+06
WAB562015*	1.0E-05						
WAB712015*	1.7E-05						
WAB712016*	2.4E-05						
NSHQ142016*	5.0E-06						
NSHQ142015*	5.0E-06	2.7E+01	3.3E-02	1.9E-01	1.7E+06	1.1E+06	6.0E+05
NSHQ142014*	7.0E-06						
	*D* at 25°C, 1 bar (m^2^ s^−1^)	1.92E-09	1.52E-09	1.19E-09	1.18E-09	1.45E-09	1.09E-09
	Reference	Cussler (2009)	Yaws (2009)	Yaws (2009)	PhreeqC	Vanýsek (1993)	Vanýsek (1993)

* Data used in calculations are from well fluid sites reported in Rempfert et al. (2017).

Of the acid forms dissolved CO_2_ (carbonic acid), formic acid, and acetic acid, dissolved CO_2_ has the highest potential flux, as shown in Figure 3B, which can be attributed to the differences in concentration among DIC, formate, and acetate. The anion forms (Figure 3C) have a more nuanced expression of potential flux, particularly in Type II fluids. There are cases where HCO_3_^−^ is more available than the anion forms of formate and acetate and vice versa, making any one of these a viable option for methanogenesis in Type II fluids. Given the orders of magnitude higher potential flux for the anion forms over the acid forms of inorganic carbon, formate, and acetate in Type II fluids, this work suggests it would be at least kinetically more favorable to use these forms if methanogens have a mechanism for taking them up. Indeed, some methanogens have been found to have transporters for formate (White and Ferry, 1992) and acetate anions (Welte et al., 2014). While transporters for HCO_3_^−^ have yet to be characterized in methanogens some bacterial chemolithoautotrophs have been found to have bicarbonate transporters (Scott et al., 2019).

#### 3.2.2 Energy availability, kJ per mole CH_4_

Energy availability does not necessarily mean faster growth or prevalence of a microbial population in a system. For example, an organism with a slow maximum growth rate may result in only a fraction of the energy available being utilized. Additionally, in a case where there is sufficient energy available, there may be energetically costly physiological stressors such as alkaline pH. Another possibility is competition for substrates, which can lower the overall experienced energy (Shock and Holland, 2007; Hoehler, 2007; Howells et al., 2022). In a system where energy is limited due to alkaline pH and the system is primarily driven by the geochemistry of the serpentinization reactions, chemical energy availability may have a more considerable impact on biology.

**Figure 4:**
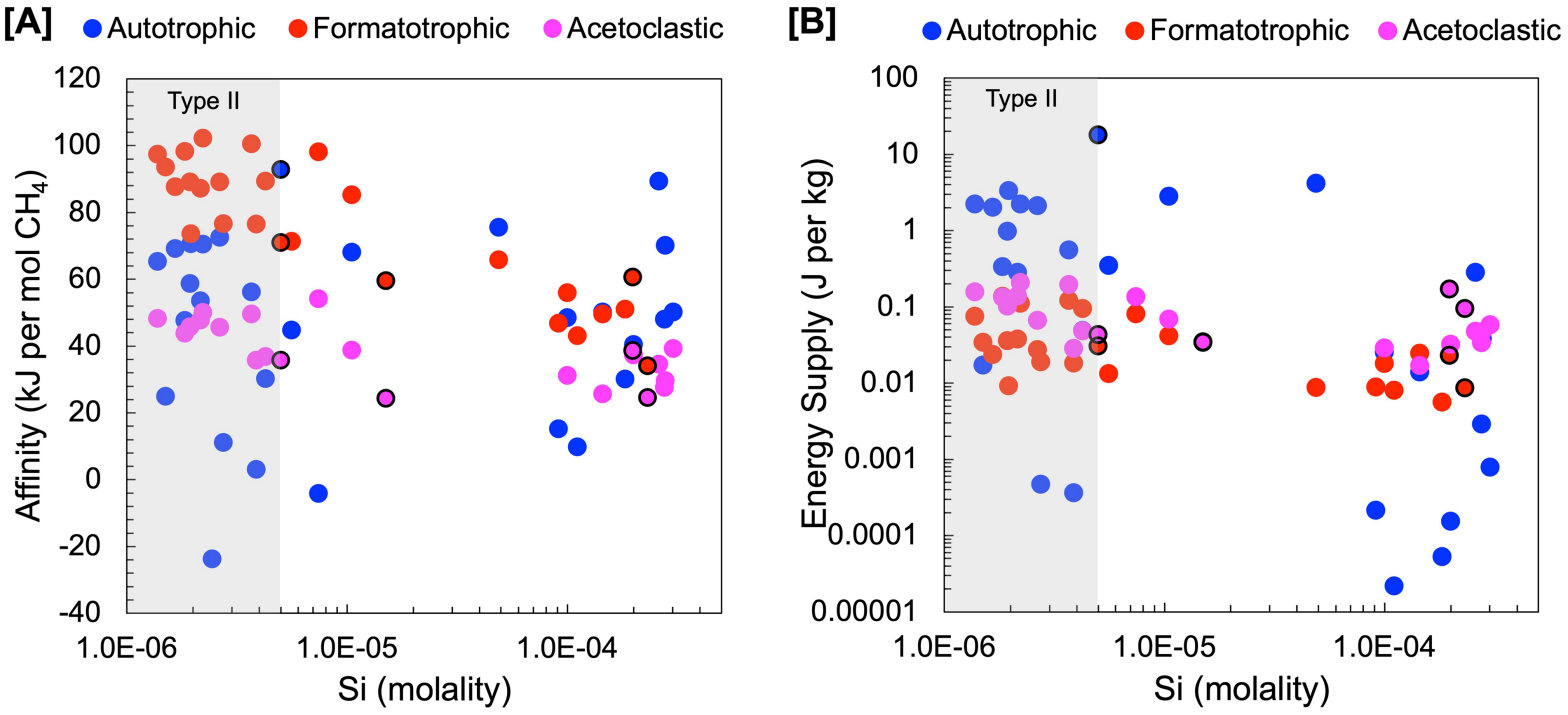
Estimations of energy availability for hydrogenotrophic (4H_2_ + HCO_3_^−^ + H^+^ ➔ CH_4_ + H_2_O), formatotrophic (4CHO_2_^−^ + H^+^ + H_2_O ➔ CH_4_ + 3HCO_3_^−^) and acetoclastic (C_2_H_3_O_2_^−^ + H_2_O ➔ CH_4_ + HCO_3_^−^) methanogenesis. Energy availability was estimated in terms of **(A)** kJ per mol of CH_4_ and **(B)** kJ per mol of kg of environmental fluid. Black outlines indicate subsurface well fluids.

Estimations of energy availability in units of kJ per mole CH_4_, shown in Figure 4A and summarized in Table 4, show that formatotrophic methanogenesis would yield the most energy in Type II fluids, followed by acetoclastic methanogenesis and hydrogenotrophic methanogenesis. While the differences in affinity among formatotrophic, acetoclastic and hydrogenotrophic methanogenesis are in some cases less than an order of magnitude, these results emphasize that formatotrophic methanogenesis is just as energy yielding as hydrogenotrophic methanogenesis, which may be surprising given the high H_2_ concentrations in serpentinized fluid. Fones et al. (2021) argue that the dependence on formate for methanogenesis by *Methanobacterium* stems from the low concentration of HCO_3_^−^ for hydrogenotrophic methanogenesis, which results in a preference for formate. That said, formate is lower in concentration than DIC, as shown in Figure 3A, which should make it a less viable substrate, especially if the acid form is used (see Figure 3B). However, given that serpentinized fluids have a uniquely low concentration of HCO_3_^−^ and 3 moles of HCO_3_^−^ are produced for every mole of CH_4_ produced by the formatotrophic pathway, the low concentration of HCO_3_^−^ as a product (and not a substrate) makes formatotrophic methanogenesis more energetically favorable than hydrogenotrophic methanogenesis. Therefore, the drive to use formate in Type II fluids may be the result of energy availability, not due to the low concentration of HCO_3_^−^ as a substrate, but as a product of formatotrophic methanogenesis.

**Table 4:** Energy availability calculations.

Sample	pH	Si	Hydrogenotrophic	Formatotrophic	Acetoclastic	Hydrogenotrophic	Formatotrophic	Acetoclastic
		Molality	kJ/CH_4_	kJ/CH_4_	kJ/CH_4_	J/kg	J/kg	J/kg
140115Z	11.33	1.38E-06	65.2	97.2	48.1	2.2E+00	7.4E-02	1.5E-01
140115X	11.398	2.22E-06	70.4	102.1	49.9	2.2E+00	1.1E-01	2.1E-01
140115Y	11.579	2.16E-06	53.5	87.1	47.7	2.8E-01	3.7E-02	1.4E-01
140114S	7.65	0.0002	40.3		37.2	1.5E-04		3.2E-02
140114U	11.355	7.43E-06	−4.2	97.9	54.0	0.0E+00	8.0E-02	1.3E-01
140114V	11.407	2.75E-06	11.0	76.6		4.7E-04	1.9E-02	
140114T	11.421	1.84E-06	47.6	98.2	43.7	3.3E-01	1.3E-01	1.3E-01
140114R	11.585	2.44E-06	−23.8			0.0E+00		
140113O	11.43	3.68E-06	56.1	100.4	49.5	5.5E-01	1.2E-01	1.9E-01
140113P	11.49	1.94E-06	58.6	89.0	45.6	9.6E-01	3.6E-02	1.0E-01
140111G	8.936	0.000276	47.9		27.5	2.9E-03		3.3E-02
140111H	10.24	0.000145	50.1	49.4	25.5	1.4E-02	2.4E-02	1.7E-02
140111I	10.87	0.0001	48.4	55.9	31.0	2.5E-02	1.8E-02	2.9E-02
140111F	11.624	1.05E-05	68.0	85.2	38.7	2.8E+00	4.1E-02	6.8E-02
140116B	7.909	0.000303	50.1		39.2	7.8E-04		5.8E-02
140116C	8.714	0.000279	70.0		29.5	3.8E-02		4.7E-02
140116D	9.104	0.00026	89.2		34.4	2.8E-01		4.7E-02
140117J	11.309	4.89E-05	75.4	65.6		4.1E+00	8.7E-03	
140117I	11.313	1.96E-06	70.4	73.5		3.3E+00	9.0E-03	
140117G	11.389	1.50E-06	24.8	93.5		1.7E-02	3.4E-02	
140117F	11.48	2.65E-06	72.4	89.0	45.5	2.1E+00	2.7E-02	6.6E-02
140117H	11.485	1.66E-06	69.1	87.6		2.0E+00	2.3E-02	
140110B	8.425	0.000183	30.0	51.0		5.2E-05	5.5E-03	
140112L	9.774	0.000111	9.6	43.0		2.2E-05	7.9E-03	
140110D	10.394	9.14E-05	15.1	46.7		2.1E-04	8.8E-03	
140112M	11.376	5.60E-06	44.7	71.3		3.4E-01	1.3E-02	
140112K	11.379	3.88E-06	2.9	76.4	35.6	3.6E-04	1.8E-02	2.8E-02
140110C	11.392	4.26E-06	30.1	89.3	36.7	4.8E-02	9.4E-02	4.7E-02
NSHQ142014*	11.4	7.00E-06						
NSHQ142015*	11.3	5.00E-06	92.8	70.8	35.6	1.8E+01	3.0E-02	4.3E-02
NSHQ142016*	11.2	5.00E-06						
NSHQ212015*	7.4	0.000782						
NSHQ3B2015*	8.4	0.000247						
NSHQ42014*	10.6	0.000198		60.5	38.5		2.3E-02	1.7E-01
NSHQ42015*	10.5	1.50E-05		59.4	24.2		3.4E-02	3.4E-02
WAB1032015*	8.2	0.00048						
WAB1032016*	8.2	0.000848						
WAB1042016*	8.5	0.00021						
WAB1052016*	8.3	4.30E-05						
WAB1882015*	8.7	0.000232		34.0	24.5		8.5E-03	9.3E-02
WAB1882016*	7.6	0.000785						
WAB552015*	9.3	6.00E-06						
WAB552016*	9.2	6.00E-06						
WAB562015*	10.6	1.00E-05						
WAB712015*	11	1.70E-05						
WAB712016*	11.1	2.40E-05						

* Data used in calculations are from well fluid sites reported in Rempfert et al. (2017).

#### 3.2.3 Energy availability, J per kg of fluid

Another way to examine energy availability is to assess the number of joules of energy that can be supplied to a microbial community in a kg of fluid in Figure 4B. This exemplifies the energy available to microorganisms in a case where 1 kg of fluid is closed off, and all energy is consumed, i.e., the reaction proceeds until the limiting reactant runs out. We can calculate this by multiplying affinity in joules per mole reaction by the concentration of the limiting reactant divided by its stoichiometric coefficient to get J per kg fluid (Equation 6). For the concentration of the limiting reactant, we summed the concentration of the total pool of chemical species the reactant is in equilibrium with; for example, for HCO_3_^−^ we summed the activities of dissolved CO_2_, CO_3_^−2^, CaHCO_3_^+^, MgHCO_3_^+^, CaCO_3_, and MgCO_3_ in addition to HCO_3_^−^ to calculate energy supply. We do this to account for the fact that as one chemical species is consumed, the system re-equilibrates for a continuous supply of the reactant until the total pool is consumed. Formate and acetate are always the limiting reactant for formatotrophic and acetoclastic methanogenesis, whereas hydrogenotrophic methanogenesis can be either H_2_-limited or inorganic carbon-limited depending on their relative concentrations.

Our results show that for energy supply (Figure 4B), hydrogenotrophic methanogenesis tends to yield the most energy, particularly in mixed fluids with the highest energy supply at a site with 99.6% serpentinized fluid. This is because inorganic carbon is the stoichiometrically limiting substrate for hydrogenotrophic methanogenesis in fluids with >70% serpentinized fluid and DIC is higher in concentration than formate and acetate in these fluids. By contrast, in fluids with <70% serpentinized fluids (high Si; mixed and Type I), formatotrophic and acetoclastic methanogenesis yield more energy per kg of fluid than hydrogenotrophic methanogenesis. In mixed and Type I fluids, hydrogenotrophic methanogenesis is stoichiometrically limited by the concentration of H_2_. Formate and acetate in these systems are higher in concentration than H_2_, and therefore, formatotrophic and acetoclastic methanogenesis yield more energy.

These results suggest that the energy available to methanogens in serpentinized fluids depends on substrate availability and how energy is experienced by the methanogen cell. If methanogens can take up HCO_3_^−^ and the anions of formate and acetate, then, based on our approximation of potential flux, these chemical species are more readily available to methanogens in Type II serpentinized fluids than dissolved CO_2_ (Figure 3C). For energy calculations, in terms of moles CH_4_ produced, formatotrophic methanogenesis is more energetically favorable than acetoclastic and hydrogenotrophic methanogenesis in Type II fluids (Figure 4A) and may explain why *Methanobacterium* preferentially uses formate in these systems. The observation that hydrogenotrophic methanogenesis can be more energetically yielding per kg fluid suggests that in a case where systems are closed off, methanogens would have a greater supply of energy for hydrogenotrophic methanogenesis and may explain why *Methanobacterium* strains in mixed fluids can use both bicarbonate (with H_2_ as an electron donor) and formate for methanogenesis as observed in Fones et al. (2021). While no acetoclastic methanogens have been previously detected in the Samail Ophiolite, energetic calculations suggest this metabolism is viable. However, given that energy availability for acetoclastic methanogenesis is comparable to hydrogenotrophic methanogenesis in Type II fluids, acetoclastic methanogens may be energetically limited. This is also reflected in an assessment of energy available in recently drilled bore-hole fluids from Oman reported in Nothaft et al. (2021b).

### 3.3 Genomic potential for methanogenesis

**Figure 5:**
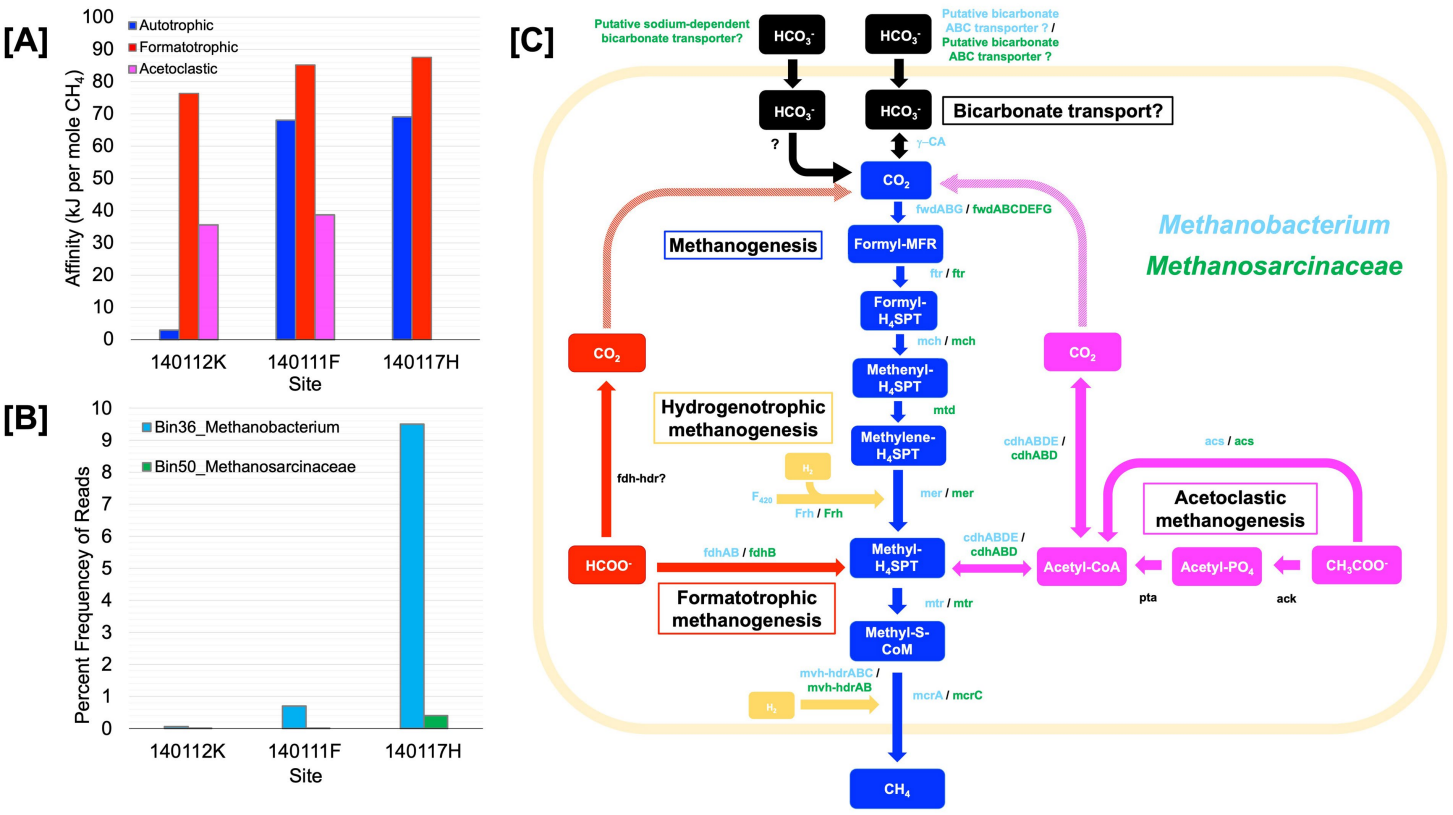
Core metabolism summary of methanogen metagenome assembled genomes. **(A)** Energy availability in kJ per mol of CH_4_ for methanogenesis reactions at shotgun metagenome sequencing sites. **(B)** Estimated percent frequency of *Methanobacterium* *Bin36* and *Methanosarcinaceae* *Bin50* at sequencing sites. **(C)** Portrays the core methanogen metabolic pathways of a *Methanobacterium* and the potential acetolactic methanogen of the genus, *Methanocarcinaceae.* Detected enzymes with >60% sequence identity of cultured methanotroph enzymes are shown in green for *Methanocarcinaceae* and orange for *Methanobacterium*. All enzymes appear next to the arrow between its substrate and product. Carbon substrates and products appear in colored boxes. The meshed arrows indicate the potential pathway in which intracellular CO_2_ is generated. Blue colored arrows and boxes indicate Methanogenesis; red indicates Formatotrophic methanogenesis; yellow indicates Hydrogenotrophic methanogenesis, purple indicates Acetoclastic methanogenesis, and black indicates the carbon concentrating mechanism. This figure is inspired by Thieringer et al. (2023). Genes: ɣ-CA, carbonic anhydrase; fwd, formylmethanofuran dehydrogenase; ftr, formylmethanofuran:H4SPT formyltransferase; mch, methenyl-H4SPT cyclohydrolase; mtd, F420-dependent methylene H4SPT dehydrogenase; mer, F420-dependent methyleneH4SPT reductase; mtr, methyl-H4SPT:coenzyme M methyltransferase; mcr, methyl-coenzyme M reductase; ack, acetate kinase; pta, phosphate acetyltransferase; acs, acetyl-CoA synthetase; cdh, carbon monoxide dehydrogenase; fdh, formate dehydrogenase; hdr, heterodisulfide reductase; mvh, methyl viologen-reducing [NiFe]-hydrogenase; frh, coenzyme F420 [NiFe]-hydrogenase; F420, coenzyme F420. Carbon compounds: CO_2_, carbon dioxide; formyl-MFR, formyl-methanofuran; formyl-H4SPT, formyl-tetrahydrosarcinapterin; methenyl-H4SPT, methenyl-tetrahydrosarcinapterin; methylene-H4SPT, methylene-tetrahydrosarcinapterin; methyl-H4SPT, methylene-tetrahydrosarcinapterin; methylene-S-CoM, 2-(Methylthio) ethanesulfonate; Acetyl-CoA, acetyl-Coenzyme A; Acetyl-PO4, acetylphosphate; CH_3_COO_−_, acetate; HCOO_−_, formate; HCO_3^−^_, bicarbonate.

To assess the genomic potential for hydrogenotrophic, formatotrophic, and acetoclastic methanogenesis, we conducted shotgun metagenome sequencing on DNA extracts from sediments of three chemically distinct Type II surface-expressed serpentinized fluids. Supplementary Figure S2 includes images of the sampling sites which are pools or streams of surface expressed serpentinized fluids. To acquire enough biological material and minimize system disruption we sampled up to 1 cm depth of sediments that underlie fluids after sampling fluid for chemical characterization. We take the bulk fluid composition that overlies the sediments to be an approximation of what microorganisms experience in the sediments. Given that the sediments are shallow due to the underlying travertine we expect fluid circulation in the sediments. One difference between overlying fluids and the sediment pore fluids may be the concentration of O_2_ the microbial communities experience, with the sediment favoring anaerobic metabolisms. Figure 5A summarizes the energetic availability of these three systems. Shotgun metagenomic sequencing revealed that, in agreement with Howells et al. (2022), *Methanobacterium* is present in surface-expressed serpentinized fluids. As summarized in Table 5, we assembled four bins classified as *Methanobacterium*. Bin36 was assembled using our “co-assembly” approach, where reads from all three sites were combined and processed to assemble bins. Bins 5, 9, and 19 were assembled from sites 140112K, 140117H, and 140111F, respectively, using our “by-site” approach where reads from individual sites were used to resolve bins. Bin9 has the highest estimated completeness (87.7%) and lowest estimated contamination (0%) of the assemblies. For the first time in Oman serpentinized fluids, in addition to detecting *Methanobacterium*, we have evidence for a potential acetoclastic methanogen of the class *Methanosarcinaceae*. We assembled two bins, one from the co-assembly approach, Bin50 (completeness 76.14%, contamination 0.65%), and one with the by-site approach, Bin11 (completeness 76.14%, contamination 2.61%), from site 140117H. We could not resolve bins for *Methanosarcinaceae* from sites 140112K and 140111F. *Methanosarcinaceae* bins were classified as JAAXQB01, which is a metagenome-assembled genome in the NCBI genome database (accession #, JAAXQB000000000) resolved from a Lost City serpentinized fluid hydrothermal vent (McGonigle et al., 2020). When examining the nearest neighbors in our classification approach (see methods), Bin50 and Bin11 only share one close relative, that being genome JAAXQB01 (see Supplementary Figure S3), which suggests serpentinized fluid *Methanosarcinaceae* are phylogenetically unique and more work must be done to characterize these novel organisms in serpentinized fluid.

**Table 5:** Assembly statistics for metagenome assemble genomes (MAGs) of methanogens and the estimated frequency of the genomes at each sequencing site.

											% Mapped Reads (Bowtie)		
Bin ID	Method	Site/s	Core methane metabolism	Taxonomy (GTDB-Tk)	Est. Compl.	Est. Contam.	GC (%)	Size (bp)	Contig (no.)	CDS	140117H, 99.9%	140112K, 99.1%	140111F, 96.9%
Bin36	Co	All	Formatotrophic/hydrogenotrophic	Family, *Methanobacteriaceae* Genus, *Methanobacterium*	80.53	0.8	35.4	883,190	227	1,051	9.5	0.06	0.7
Bin9	By site	140117H	Formatotrophic/hydrogenotrophic	Family, *Methanobacteriaceae* Genus, *Methanobacterium*	87.73	0	35.3	1,123,095	265	1,358	12.8	0.08	0.9
Bin19	By site	140111F	Formatotrophic/hydrogenotrophic	Family, *Methanobacteriaceae* Genus, *Methanobacterium*	83.04	1.07	35.2	1,245,005	298	1,539	7.8	0.05	0.5
Bin5	By site	140112K	Formatotrophic/hydrogenotrophic	Family, *Methanobacteriaceae* Genus, *Methanobacterium*	59.11	0.4	35.3	732,008	288	1,046	12.5	0.08	1.0
Bin50	Co	All	Acetoclastic/hydrogenotrophic	Family, *Methanosarcinaceae* Genus, JAAXQB01	76.14	0.65	33.7	898,440	162	1,006	0.4	0.01	0.01
Bin11	By site	140117H	Acetoclastic/hydrogenotrophic	Family, *Methanosarcinaceae* Genus, JAAXQB01	76.14	2.61	33.9	949,132	165	1,054	0.4	0	0.02

We assessed the relative frequency of the methanogen bins reported in Table 5, focusing on Bin36 (*Methanobacterium*) and Bin50 (*Methanosarcinaceae*) (co-assembled MAGs) in Figure 5B. Among the three sites, *Methanobacterium* *Bin36* is the most abundant at site 140117H, which is the most H_2_-rich and has the highest energy available in terms of kJ per mole CH_4_ for formatotrophic and hydrogenotrophic methanogenesis. Bin36 is lowest in abundance at site 140111K, which has the least energy available for hydrogenotrophic and acetoclastic methanogenesis. *Methanosarcinaceae* Bin50 has the highest frequency at site 1401117H, although it is incredibly low (0.4%) when compared to the frequency of *Methanobacterium* Bin36 (9.5%). While we could not characterize energy availability for acetoclastic methanogenesis at this site due to acetate being below detection, another Type II fluid site in the same sampling location as 1401117H has comparable energy availability as the other two sites in this analysis, 140111K and 140111F. Therefore, the energy availability for acetoclastic methanogenesis may not be a strong determinant for the occurrence of *Methanosarcinaceae* in serpentinizing systems.

Given the estimated completeness of the genomes and the number of contigs that did not pass the binning process at each site (see methods), we take these genomes to be draft genomes. However, valuable insights into methanogen metabolisms can be gained from these assemblies. Figure 5C summarizes the core metabolisms of the *Methanobacterium* and *Methanosarcinaceae* bins. As Figure 5C illustrates, we see the genomic potential for formatotrophic methanogenesis by *Methanobacterium* in surface-expressed Type II fluids with the presence of formate dehydrogenase (fdh). *Methanobacterium* genomes also show evidence for hydrogenotrophic methanogenesis with the presence of methyl viologen reducing hydrogenases (mvh) and CoB-CoM heterodisulfide reductase (hdr) as well as coenzyme F420-reducing hydrogenase (Frh) and membrane bound NiFe hydrogenase (see Supplementary Table S5). As Thieringer et al. (2023) observed, *Methanobacterium* in our study may be able to use acetate as a carbon source with the presence of acetyl-CoA synthetase (acs) and carbon monoxide dehydrogenase (cdh).

In surface-expressed Type II *Methanobacterium* genomes, we also see potential for uptake and utilization of HCO_3_^−^ as a carbon source for hydrogenotrophic methanogenesis. We detect putative ATP-dependent ABC nitrate/sulfonate/bicarbonate transporter in all three *Methanobacterium* bins. While the exact function of this transporter is unknown, it is not out of the realm of possibilities for chemolithotrophs to have functional bicarbonate transporters (Scott et al., 2019), however, bicarbonate transporters have yet to be characterized in methanogens. If *Methanobacterium* has functional bicarbonate transporters, once bicarbonate is transported, it is possible that the gamma carbonic anhydrase detected in *Methanobacterium* bins in this study can convert HCO_3_^−^ to CO_2_ for hydrogenotrophic methanogenesis. This process may require some form of a DIC concentrating mechanism, like a carboxysome (an organelle-like structure that traps CO_2_ and concentrates carbon in bacteria), but whether or not a carboxysome is required is dependent on the DIC flux required at the in-situ growth rate. Carbon concentrating mechanisms have yet to be observed in methanogens and remains a point of exploration, particularly for serpentinized fluids. It is worth noting that carbonic anhydrase was not detected in subsurface *Methanobacterium* genomes characterized in Fones et al. (2021) and Thieringer et al. (2023), making this a unique attribute of surface-expressed serpentinized fluid *Methanobacterium*.

Despite the low estimated completeness of the *Methanosarcinaceae* bins, examining the core metabolism of these genomes reveals that these methanogens have the functional potential to carry out acetoclastic methanogenesis and hydrogenotrophic methanogenesis. The bins of *Methanosarcinaceae* lack the genes that code for acetate kinase (ack) and phosphate acetyltransferase (pta), which together can convert acetate to acetyl-CoA, however they have acetyl-CoA synthetase (acs) which directly converts acetate to acetyl-CoA. Acetoclastic methanogens of the genus *Methanothrix* are known to have acs, which has a higher substrate affinity for acetate than methanogens of the genus *Methanosarcina*, which use ack and pta for acetate activation (Stams et al., 2019). It may be that the overall low organic carbon content of serpentinizing systems selects for acetoclastic methanogens with a high substrate affinity for acetate. It is worth noting that the activation of acetate by acs requires two ATP (Stams et al., 2019). The energetic cost to make 1 ATP from 1 ADP is ~45 kJ per mol CH_4_ (Thauer et al., 1977). Based on our affinity calculations there is on average 38 ± 9 kJ per mole CH_4_, which is only enough energy for production of 1 ATP and therefore not enough for acetate activation. However, the bins shown evidence for H_2_ utilization. If both pools of energy (hydrogenotrophic and acetoclastic) are available to acetoclastic methanogens, then there is more than enough energy for acetate activation. Indeed, some acetoclastic methanogens have been shown to also use H_2_ and CO_2_ (Welte and Deppenmeier, 2014). It is also worth noting the complete pathway for autotrophy in the *Methanosarcinaceae* bins. It is possible that CO_2_ can be supplied through acetate uptake and conversion to CO_2_ (with acetyl-CoA as intermediate) by carbon monoxide dehydrogenase (cdh). This may explain why the frequency of Bin11 is highest at 140117H, which has the highest energy availability for hydrogenotrophic methanogenesis as the electron donor among the three sequencing sites.

**Figure 6:**
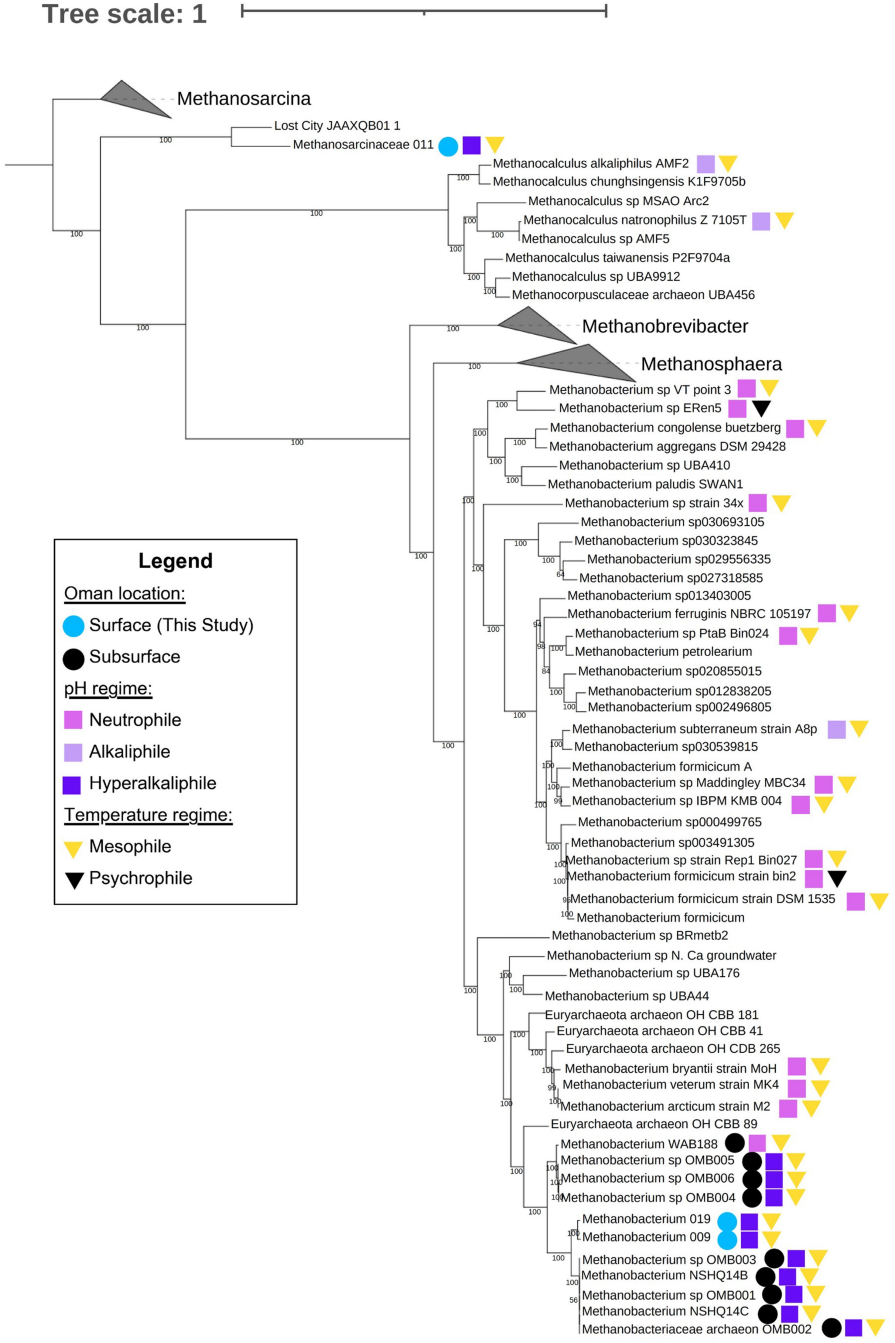
Maximum likelihood phylogenomic tree of *Methanobacterium*, *Methanosarcinaceae* and related taxa constructed via GToTree using 76 archaeal marker genes identified by the program. Within GToTree, IQ-TREE was called in order to generate the tree. The program was run with 1,000 bootstrap replicates; bootstrap confidence estimates (out of 100) are shown at nodes. We included 11 genomes from Samail Ophiolite Methanobacterium and 18 genomes from other methanogenic archaea which had satisfactory metadata and genome completeness. Also included were GTDB representative genomes from the genera *Methanobacterium*, *Methanosphaera*, *Methanobrevibacter*, and *Methanosarcina*, as well as genomes identified by GTDB-Tk - v2.3.2 as “close relatives” of *Methanocalculus natronophilus*, *Methanobacterium congolense* buetzberg, and the Oman MAGs isolated by Thieringer et al., 2023 and in this study. Genomes for which relevant metadata was available are marked on the tree. Circles indicate genomes from surface (light blue) and subsurface (teal) environments among methanogens taken from the Samail Ophiolite in Oman. Squares indicate pH affinity, neutrophile (mauve), alkaliphile (lavender), or hyperalkaliphile (dark purple), of all relevant strains; and triangles indicate temperature affinity, mesophile (yellow) or psychrophile (black), of all relevant strains. Neutrophiles were defined as organisms which, whether through environmental or culture-derived data, were determined to thrive between pH 6.5 and 8.5 but no higher; alkaliphiles were defined as those which thrived between pH 8.5 and 10 but no higher; hyperalkaliphiles were those which thrived at pH 10 and above. Similarly, psychrophiles were defined as organisms which, whether through environmental or culture-derived data, were determined to thrive below 20°C; mesophiles were those which thrived between 20 and 50°C but no lower. More information on these genomes and the metadata used to label this tree can be found in Supplementary Table S5.

We carried out a phylogenomic analysis to compare our *Methanobacterium* genomes to those published in Fones et al. (2021) and Thieringer et al. (2023). The resulting maximum likelihood phylogenomic tree shows the pattern of macroevolutionary relationships between Samail Ophiolite *Methanobacterium* and other methanogens, including other *Methanobacterium* species, as well as *Methanosphaera, Methanobrevibacter, Methanocalculus,* and the *Methanosarcinaceae.* Bootstrap values at nodes indicate the percentage of replicate trees in which a given node was observed; these were overwhelmingly close to 100, as can be seen in Figure 6. Genomes for which there was reliable environmental data available were labeled according to environment type. Colored squares were used to indicate pH affinity. The highest observed optimum or environmental pH for each genome was used to assess whether it fell into the following categories: neutrophile: pH 6.5 to 8.5; alkaliphile: pH 8.5 to 10; hyperalkaliphile: pH 10 and above. Colored triangles indicate temperature affinity. Psychrophiles were those with their lowest point in optimum or observed temperature below 20°C; all others were mesophiles. Among genomes from the Samail Ophiolite, colored circles indicate whether genomes were found at the surface or subsurface. The root of the tree is based on Thieringer et al. (2023). An unrooted tree is shown in Supplementary Figure S4.

This analysis shows that *Methanobacterium* bins from surface-expressed fluids share a common ancestor with Type II *Methanobacterium* from the subsurface (Figure 6). The phylogenomic relationship and core metabolism annotation suggest that like Type II *Methanobacterium* from subsurface well fluids (Fones et al., 2021) *Methanobacterium* from the surface may be able to use formate as an electron donor and have maintained functional capability for hydrogenotrophic methanogenesis. The phylogenomic tree also reveals that *Methanobacterium* from Oman form their own clade distinct from neutrophilic *Methanobacterium*. There is one *Methanobacterium* cultivar considered to be an alkaliphile, *M. subterraneum*. However, it is in a clade with other neutrophilic *Methanobacterium* genomes. This suggests that adaptations to serpentinized fluids may extend beyond adaptation to alkaline pH alone. For *Methanobacterium*, this may be attributed to carbon limitation, as described in Fones et al. (2021). The *Methanosarcinaceae* Bin11 and Lost City MAG (JAAXQB01) form their own distinct clade and appear to share a common ancestor with other *Methanosarcina* (see unrooted tree in Supplementary Figure S4). The new serpentinizing system clade are closely relately to methanogens of the genus *Methanocalculus*, some species of which are alkaline soda lake methanogens that can carry out hydrogenotrophic methanogenesis and require acetate for growth (Sorokin et al., 2015). This suggests that alkaline pH may be a selective pressure for evolution of acetoclastic and acetate-utilizing methanogens.

Overall, examining methanogen genomes in surface-expressed Type II fluids shows that hydrogenotrophic methanogenesis with HCO_3_^−^, formatotrophic methanogenesis, and acetoclastic methanogenesis are genomically possible, even co-occurring in these systems. The genomic potential for formatotrophic methanogenesis by *Methanobacterium* combined with energetic calculations supports the hypothesis that there may be an energetic drive for *Methanobacterium* to carry out formatotrophic methanogenesis, despite the high concentration of H_2_ available for hydrogenotrophic methanogenesis. H_2_ may may act as an additional electron donor for methanogenesis by *Methanosarcinaceae* in serpentinizing systems after acetate is converted to CO_2_ by carbon monoxide dehydrogenase. The first-time detection of genomes in the Samail Ophiolite classified as *Methanosarcinaceae* and their phylogenomic similarity to *Methanosarcinaceae* genomes from Lost City suggest that potential acetoclastic methanogens in serpentinizing systems are unique and beg for deeper characterization.

## 4 Conclusion

In this study, we found that in the most reduced, hyperalkaline, H_2_-rich serpentinized fluids in the Samail Ophiolite, formatotrophic methanogenesis yields more energy than hydrogenotrophic and acetoclastic methanogenesis in terms of kJ per mole CH_4_ produced. While these fluids are H_2_-rich, they are also depleted of inorganic carbon. Consequently, formatotrophic methanogenesis, a reaction that produces 3 moles of inorganic carbon per mole of CH_4_ produced, can be more energetically favorable than hydrogenotrophic methanogenesis with H_2_ as the electron donor. This may explain why formatotrophic *Methanobacterium* from Oman characterized in subsurface Type II fluids in Fones et al. (2021) and possibly surface-expressed *Methanobacterium* characterized in this study may preferentially use formate over bicarbonate in Type II fluids. That said, in terms of J per kg of fluid, hydrogenotrophic methanogenesis yields more energy than formatotrophic and acetoclastic methanogenesis, especially in mixed fluids where H_2_ is not stoichiometrically limited, and there is a greater supply of inorganic carbon due to fluid mixing. While mixed fluids pose a problem to methanogens at the surface due to the influence of O_2_ (Howells et al., 2022), the energy available for hydrogenotrophic methanogenesis in mixed subsurface fluids may explain the ability of *Methanobacterium* (genome Type I) to use inorganic carbon and formate for methanogenesis as reported in Fones et al. (2021).

Our work finds that acetoclastic methanogenesis is energetically favorable in serpentinized fluids in Oman. However, previous studies on the microbial communities in the Samail Ophiolite failed to detect acetoclastic methanogens. In this study, we assembled for the first time a genome classified as belonging to the acetoclastic methanogen family, *Methanosarcinaceae*. Based on phylogenomic analyses, this MAG has only one close relative, a genome of an acetoclastic methanogen of the family *Methanosarcinaceae*, JAAXQB01, resolved from the Lost City serpentinizing hydrothermal vent field (McGonigle et al., 2020). The unique nature of these genomes inspires deeper characterization of a potentially novel methanogen group.

Overall, this work stresses the importance of considering both substrate availability and energy availability when examining factors that influence methanogen distribution and evolution on Earth. For the same reason that hydrogenotrophic methanogenesis is an energetically costly process in serpentinized fluids, formatotrophic methanogenesis is energetically favorable and circumvents the cost of utilizing CO_2_ in an inorganic carbon-limited environment. As we look beyond Earth and consider what life may be supported on Enceladus or Europa, this study suggests a broader suite of carbon substrates could support life like methanogens on these moons, particularly given that formate can be produced abiotically under hydrothermal, serpentinizing conditions.

## Data Availability

The original contributions presented in the study are publicly available. This data can be found here: https://www.ncbi.nlm.nih.gov/sra, under the BioProject number PRJNA1207711. The metagenome-assembled genomes presented in this study will be made available by the authors without undue reservation upon request.

## Footnotes

^1^ https://worm-portal.asu.edu

^3^ https://www.kbase.us

^4^ http://www.bioinformatics.babraham.ac.uk/projects/fastqc/
