# Supplementary figures and images for: Energetic and genomic potential for hydrogenotrophic, formatotrophic, and acetoclastic methanogenesis in surface-expressed serpentinized fluids of the Samail Ophiolite
Source: Front Microbiol. 2025 Jan 31;15:1523912. doi: 10.3389/fmicb.2024.1523912 (PMC11826062; doi:10.3389/fmicb.2024.1523912)

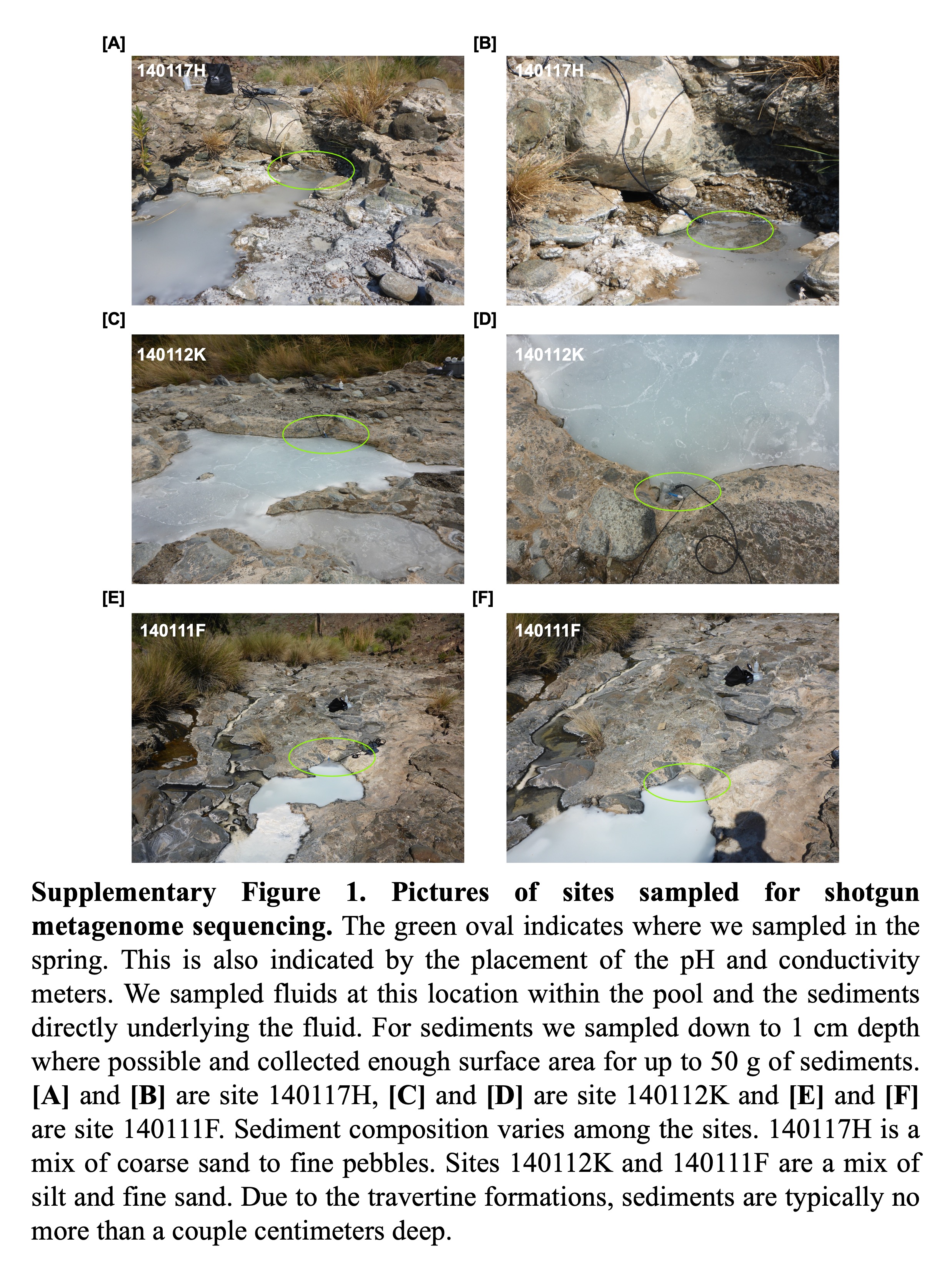

Supplement: Supplementary file 6 [file Image_1.jpeg]

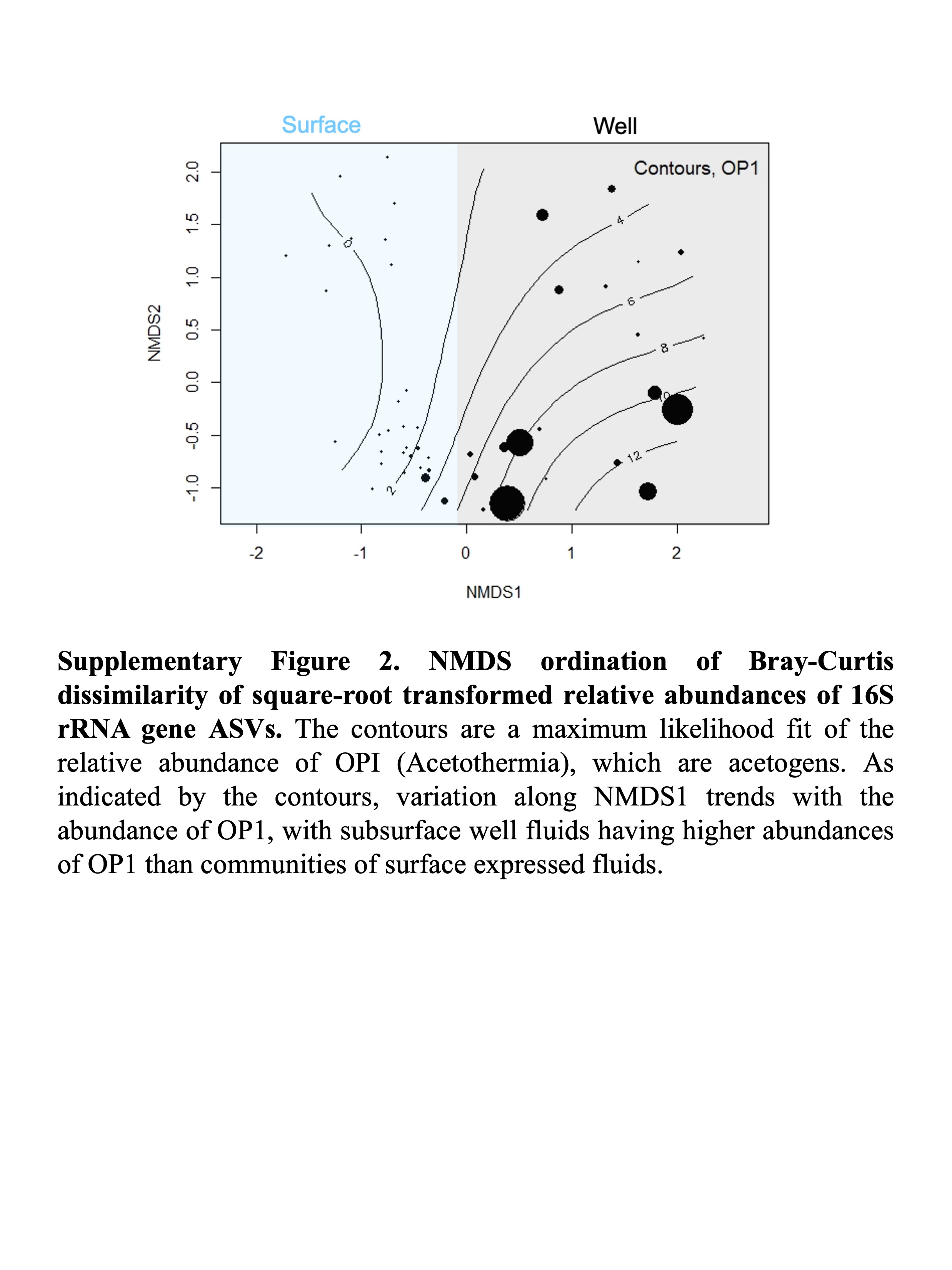

Supplement: Supplementary file 7 [file Image_2.jpeg]

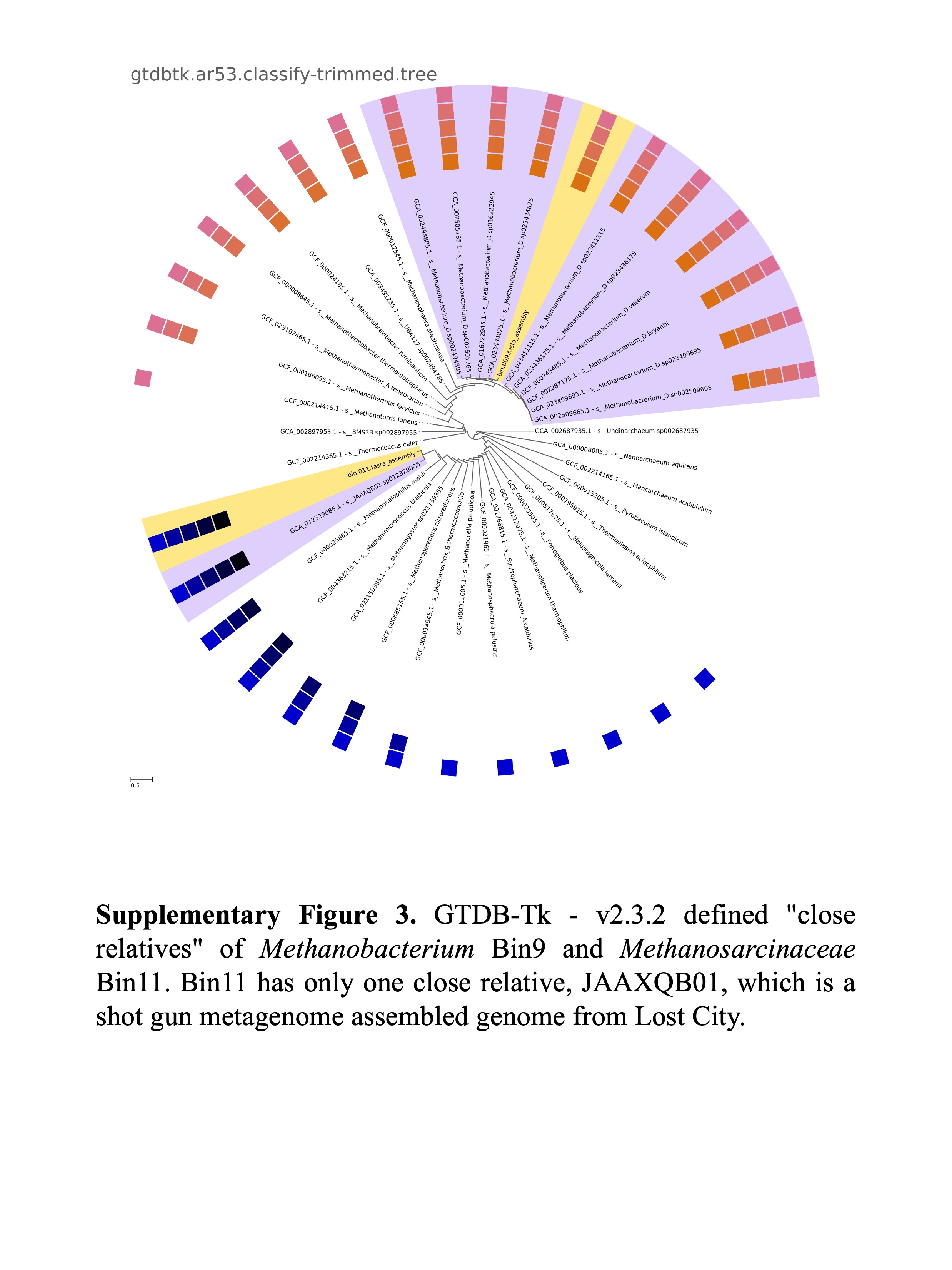

Supplement: Supplementary file 8 [file Image_3.jpeg]

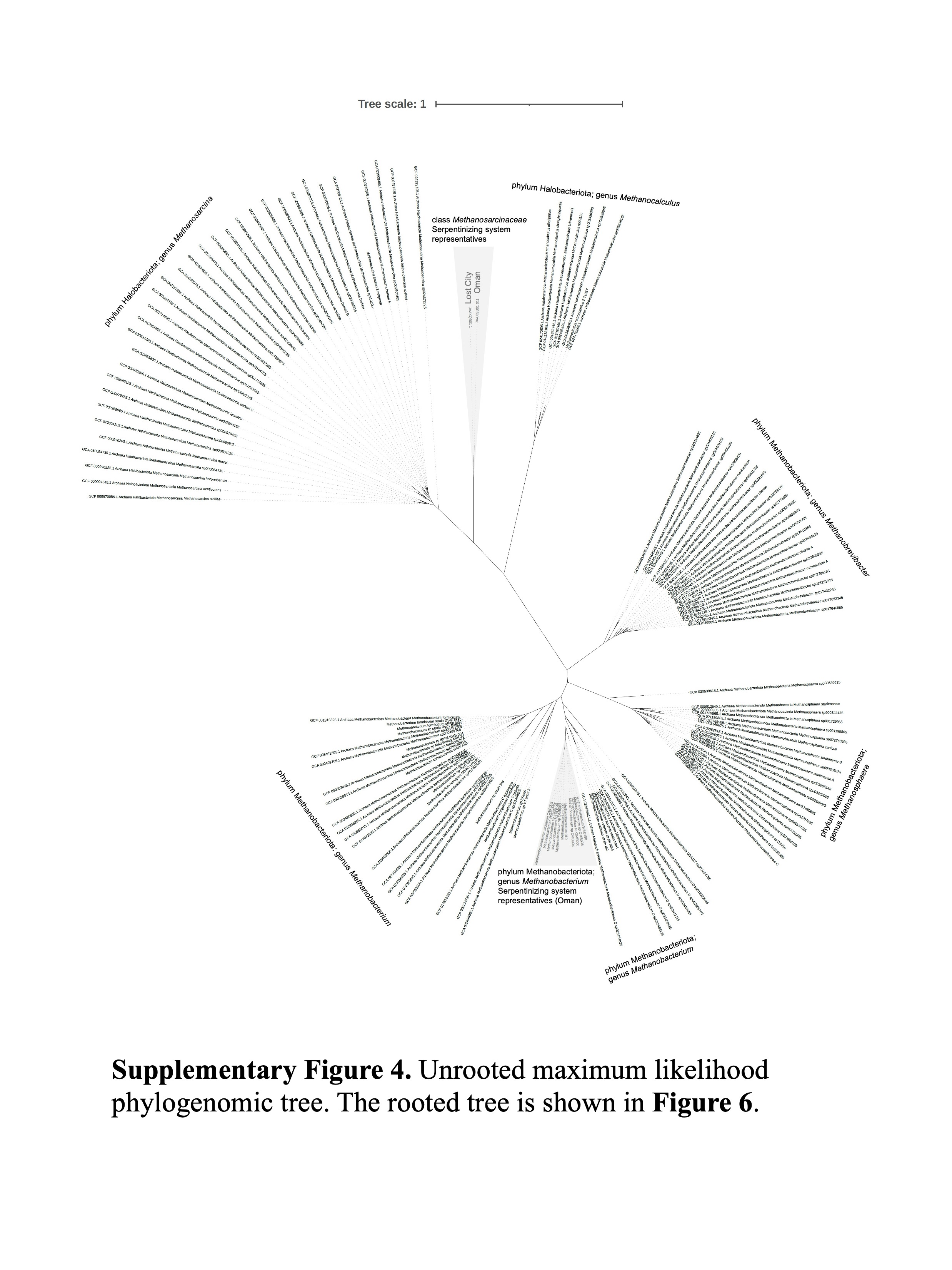

Supplement: Supplementary file 9 [file Image_4.jpeg]
